# Relationships between changing communication networks and changing perceptions of psychological safety in a team science setting: Analysis with actor-oriented social network models

**DOI:** 10.1371/journal.pone.0273899

**Published:** 2022-08-31

**Authors:** Jinwen Luo, Minjeong Jeon, Minho Lee, Eric Ho, Angela Fidler Pfammatter, Vivek Shetty, Bonnie Spring

**Affiliations:** 1 University of California, Los Angeles, Los Angeles, California, United States of America; 2 Northwestern University, Evanston, Illinois, United States of America; University of Greenwich, UNITED KINGDOM

## Abstract

A growing evidence base suggests that complex healthcare problems are optimally tackled through cross-disciplinary collaboration that draws upon the expertise of diverse researchers. Yet, the influences and processes underlying effective teamwork among independent researchers are not well-understood, making it difficult to fully optimize the collaborative process. To address this gap in knowledge, we used the annual NIH mHealth Training Institutes as a testbed to develop stochastic actor-oriented models that explore the communicative interactions and psychological changes of its disciplinarily and geographically diverse participants. The models help investigate social influence and social selection effects to understand whether and how social network interactions influence perceptions of team psychological safety during the institute and how they may sway communications between participants. We found a degree of social selection effects: in particular years, scholars were likely to choose to communicate with those who had more dissimilar levels of psychological safety. We found evidence of social influence, in particular, from scholars with lower psychological safety levels and from scholars with reciprocated communications, although the sizes and directions of the social influences somewhat varied across years. The current study demonstrated the utility of stochastic actor-oriented models in understanding the team science process which can inform team science initiatives. The study results can contribute to theory-building about team science which acknowledges the importance of social influence and selection.

## Introduction

A growing evidence base indicates that finding solutions to complex scientific problems is enhanced when diverse experts collaborate to develop cross-disciplinary solutions. Scientists who practice interdisciplinary team science benefit as well. Especially in the life and physical sciences [1], they tend to secure more scientific funding [2] and produce higher-impact publications than their less interdisciplinary peers. Nonetheless, effective team collaborations are difficult to actualize [3]. A greater understanding of the processes by which effective transdisciplinary teams communicate and collaborate could facilitate the formation of teams and the facilitation of processes that optimize scientific discovery [4–6].

Research on the Science of Team Science (SciTS) suggests that social interactions within teams impact team effectiveness [7–9], and teams are more productive when greater familiarity and social cohesiveness exist among team members [10]. Although sociodemographic similarities promote network ties within teams [11], homogeneous teams do not necessarily outperform heterogeneous teams on creative and intellectual tasks [12]. Team members’ psychological attributes (i.e., attitudes, motivation, and emotions) also consistently impact team performance and outcomes [13, 14]. For example, members who feel safe when they interact with other team members may communicate in a more positive and respectful manner. Those communications, in turn, may create a positive feedback loop that reinforces positive communications and feelings of safety within the team, enhancing participants’ motivation to work together. As a result, teams whose members have strong perceptions of psychological safety may collaborate better and produce higher quality outcomes than those whose members feel less psychologically safe with engaging with others on their team [15, 16]. However, the dynamics of how team members’ psychological states change in relation to their communication patterns have rarely been explored in the SciTS literature.

To close this knowledge gap, we studied whether team members’ communications evolved in parallel with changes in their perceptions of psychological safety when teams worked to develop a capstone project during the NIH-sponsored mHealth Training Institute (mHTI). The annual mHTI assembles selected scholars from diverse disciplinary backgrounds (e.g., computer science, engineering, medicine, public health, psychology) and different institutions to collaborate on developing a research proposal to design and evaluate a mobile health (mHealth) solution to a critical healthcare problem. The mHTIs provide an attractive testbed to study the formation and coevolution of transdisciplinary teams because their disciplinarily and geographically diverse scholars bring different knowledge bases, norms, and perspectives on collaboration to their capstone projects. Solving a real-world problem requires achieving conceptual integration across disparate expertise through active social and intellectual interactions [17]. The need to socially integrate with new collaborators from different backgrounds and disciplinary frameworks in order to develop a competitive proposal can stimulate psychological insecurity, particularly in the context of the brief, intensive week-long mHTI schedule. Developing communications that foster solid psychological safety, which is needed to fully elicit all participants’ expertise, is a team science process that should contribute to a high-quality capstone product.

Our paper explores how individuals’ communication networks form and develop in an interdisciplinary training program, how their perceptions of psychological safety evolve in relation to their team communication patterns, and how the two processes interact with each other during the program. Through the novel application of stochastic actor-oriented models (SAOMs) [18, 19] in the context of initiating an interdisciplinary team science collaboration, we investigate the dynamics between the evolution of participants’ communication networks and their perceptions of psychological safety. Understanding the processes by which team states co-evolve could help foster an optimal climate for interdisciplinary collaborations and cultivate new norms for team science-based training. Furthermore, exploring the dynamic nature and process of team collaborations allows for a creative application of stochastic actor-oriented models to the study of team science processes.

## Materials and methods

### Data

The mHealth Training Institute (mHTI) collected program and team process data during the week-long program. Each multidisciplinary team was charged with developing a capstone project that designed and evaluated a mobile health solution to a real-world healthcare problem. Each team gave a formal presentation on their project on the last day of the mHTI, and their work was evaluated by the faculty mentors. During the week-long institute, participants spent five hours each day working with other members of their team on their capstone project. All participants came together for the common lectures and networked with others during the group breakfasts, lunches, and dinners. Surveys about participants’ self-reported communication networks and feelings, beliefs, and attitudes, including team psychological safety, were administered at the end of mHTI days 1, 3, and 5.

#### Participants

We analyzed the data from the 2017, 2018, and 2019 mHTIs. A total of 29 scholars participated in 2018 and 2019, and 35 scholars participated in the 2017 program. Each mHTI selected scholars from a pool of more than 300 applicants. Scholars, who ranged from early to later career, were grouped into four main disciplinary categories: Medicine/Nursing (MED), Public Health/Others (OTH), Psychology (PSY), and Computer Science/Engineering/Data Science (CS). Admitted candidates were assigned to one of five teams for the training institute, balancing the representation of disciplinary backgrounds across teams.

#### Project-based conversation network

On the first, third, and fifth days of the program in all years, scholars were asked to report those with whom they discussed their project since the prior assessment. For each data collection point (each day for each cohort), an *N* by *N* adjacency matrix *A* was created to indicate the interpersonal nominations of project-based communication partners (where *N* is the number of scholars). The rows of the matrix indicate the scholar indexed by *i* while the columns indexed by *j* are the same list of scholars in the same order. If scholar *i* talked to scholar *j*, the *i*-th row and *j*-th column element of the adjacency matrix takes value 1 (and 0 if not). Throughout the paper, we use the term ‘ego’ to indicate scholar *i* who sent a conversation (tie) and ‘alter’ to indicate scholar *j* who received a conversation, consistent with the social network literature [20]. The diagonal consists of zeros (*A*_*i*,*i*_ = 0) because there is no self-nomination in the communication network. The upper diagonal part of the matrix can be different from the lower diagonal counterpart (*A*_*i*,*j*_ ≠ *A*_*j*,*i*_) because scholar *i* talking to scholar *j* does not necessarily indicate that scholar *j* talked to scholar *i*; that is, there is a direction in the recorded conversations. For instance, while scholar *i* might consider the conversation important enough to report, scholar *j* might not feel the same. Thus, the networks being analyzed (represented with the described adjacency matrices) are *directed networks*. We created three adjacency matrices that reflect scholar communications over three time points (day1, day3, and day5) during the mHTI program for each of three years (2017, 2018, and 2019). Our analysis is concerned with changes in the three adjacency matrices (day1, day3, and day5) per year.

Each adjacency matrix contains relational information among the scholars in the communication network. Therefore, as an *actor* in the network, each scholar has their network properties besides individual attributes. For example, each scholar can nominate and be nominated by a different number of scholars as conversation partners. Scholars who send out many nominations can be considered *active* actors, while *popular* actors are those who received many nominations by other scholars. If both members of a scholar pair nominated each other as conversation partners, a mutual edge is created, indicating reciprocity in the network. Details on these and other network measures utilized in this paper are provided in S1 Table.

Actors’ network attributes can evolve over time along with their individual attributes. We are interested in understanding how the evolution of a project-based professional communication network interacts with the evolution of scholar participants’ psychological safety during the team science process.

#### Perceptions of psychological safety

The end-of-day survey asked scholars about their *perceptions of team psychological safety*, defined as “a shared belief held by members of a team that the team is safe for interpersonal risk-taking” [16]. This construct was measured with a seven-item scale that has been widely used in the team science literature [16, 21–23]. The item composition of the scale is summarized in Table 1. Each item was rated for the degree of agreement-disagreement using a 7-point Likert scale. Higher values on the scale indicate more positive feelings about psychological safety in teamwork. Three items (item 1, 3, and 5) were negatively worded, and thus reverse-coded. To create a composite score, we first dichotomized each item with the cut score of 6 (indicating a high level of endorsement), and then counted the number of endorsed items. The resulting composite score ranged from 0 to 7. The internal consistency of the scale, measured with Cronbach’s alpha, was satisfactory, ranging from 0.69 to 0.88 across the nine datasets being analyzed (three years times three days per year).

**Table 1:** Item composition of the perceptions of team psychological safety scale.

Measure	Items
Team Psychological Safety	1. If I make a mistake in this team, it is held against me.*
	2. Members of this team are able to bring up problems and tough issues.
	3. People on this team sometimes reject others for being different.*
	4. It is safe to take a risk in this team.
	5. It is difficult to ask other members of this team for help.*
	6. No one on this team would deliberately act in a way that undermines my efforts.
	7. Working with members of this team, my unique skills and talents are valued and utilized.

* denotes reversely coded.

^a^ The scale is developed and validated by Edmondson [16].

### Stochastic actor-oriented model

To examine the coevolution of the scholars’ communication networks and their perceptions of team psychological safety, we applied the stochastic actor-oriented model (SAOM) [24, 25]. The SAOM enables us to examine the changes in scholars’ communication networks and changes in their perceptions of team psychological safety [19]. The submodel for the communication network estimates the likelihood that scholars’ communication ties with other scholars form or persist over time given individuals’ attributes (including their perceptions of team psychological safety) and the communication network structure. The perception submodel predicts the scholar’s evolution in the perception of team psychological safety, which is the outcome variable of this submodel, given their individual attributes and other scholars’ perceptions of team psychological safety with whom they have communicated. That is, the submodel for the communication network involves changes in perceptions of psychological safety, while the submodel for the perception involves changes in the communication network; hence, we can examine interactions between the changes in the communication network and the perception of team psychological safety.

SAOM analysis of psychological attributes such as team psychological safety has been seen in recent literature. For example, Igarashi and Hirashima [26] tested generalized trust in the social selection process with SAOM. Their model was applied to four waves of advice and personal discussion networks among first-year undergraduates. In their research, generalized trust is a psychological trait measured by six items. Another example can be found in Sadewo et al. [27], where two latent psychological traits (psychological adjustment and sociocultural adjustment) were analyzed with SAOM to test whether cross-cultural adjustment is a result of social selection or social influence among international graduate students.

Perception of team psychological safety, the key interest of our study, can be viewed as one of the consequences of team interactions. Team members can observe the status and any changes in the manifestations of the psychological safety of other team members and may be influenced by them and/or take actions to influence others’ psychological safety. For example, a team member *i* may observe that another member *j* feels psychologically unsafe within the team. The member *i* may empathize with his/her peer *j* and discuss the feeling of unsafety within the team. On this ground, the use of the SAOM framework for the perception of team psychological safety is reasonable and justifiable.

SAOM offers a unique perspective to understand mechanisms behind homophily, a common phenomenon observed in human networks, which indicates that individuals tend to associate with others who are similar to themselves. Selection and influence are two major mechanisms that underlie homophily and that are also often observed in team collaborations. In the mHTI’s team collaboration context, social selection assumes that scholars tend to have conversations with others based on their similarity in particular individual attributes that are observed (e.g., gender) or latent (e.g., psychological safety). We hypothesize that a specific kind of social selection can occur, such that scholars communicate more frequently with others who have similar levels of perception of team psychological safety. For example, scholars may express different levels of engagement in team communication activities based on their levels of team psychological safety. Scholars in turn may choose to communicate with others depending on different engagement levels expressed by their peers. Our social selection hypothesis can be tested by examining the presence and magnitude of the similarity effects measured in the network change submodel. To test the described social selection process, we control for other types of homophily, such as gender and discipline homophily. Social influence, on the other hand, assumes that the scholars’ perceptions of team psychological safety are influenced by the colleagues whom they have communicated with during the mHTI program. The social influence hypothesis can be tested by examining the average similarity effects between egos and alters in the perception change submodel. If social influence were present, a scholar’s perception of team psychological safety would be influenced by other scholars’ team psychological safety whom they communicated with.

The SAOMs were estimated using the RSiena package in R [28]. The team’s psychological safety was coded into ordinal measures as described earlier, which are required for SAOM analysis [25]. Missing data on network and team psychological safety were imputed following the standard SAOM imputation procedure [29]. RSiena simulates and updates networks, target behavior variables, and attributes iteratively to minimize differences between simulation and observed target statistics for those attributes. We tested whether the estimated models converged adequately based on the overall maximum convergence ratios and the t-statistics for deviations from targets (i.e., t-ratio). Overall, a maximum convergence ratio below the threshold of 0.25 and the t-ratio below the criterion of 0.10 indicate adequate model convergence [25]. Further, we evaluated the goodness-of-fit of the estimated models based on important target statistics, including outdegrees, indegrees, distributions of team psychological safety perceptions, and triad census [30].

#### Model specification

We constructed four SAOMs to examine the evolution of mHTI scholars’ network-perception during the program per year. S1 Table provides mathematical details of the terms included in the models.

*Model 1*: *Basic model*. The basic model explores how the scholars’ communication ties form and persist and how their perceptions change during the mHTI. The network and perception change submodels include two rate parameters for day 1 to day 3 (*rate 1*), and day 3 to day 5 (*rate 2*), respectively. The rate parameters in the submodels indicate the extent to which expected network and perception changes are likely to occur between different observed time points (i.e., how many opportunities an actor has for changing ties and perceptions between two time points). The rate parameters control for the volume of communication and perception changes between the time points. Positive and significant rate parameters indicate successive and significant expected number of opportunities for changes in communication ties and perception of psychological safety between the two time points. It is important to note that rate parameters are expected opportunities for changes in the network or perceptions, meaning that observed changes between two adjacent time points can be lower than the rate parameters due to two possibilities: (a) actors had an opportunity to change their behavior but decided not to change the behavior; and (b) actors changed their behavior twice, such that, for instance, they once increased the behavior and then decreased it; in this case, they returned to their initial states before the next wave [18].

The network change submodel includes a set of network structure parameters to capture the overall structure of the project-based conversation network. The following network structure parameters were considered in the basic submodel [25]:

- *outdegree (density)* measures the overall density of communication ties within the network, related to how likely a tie is to occur after controlling for all other effects in the model.
- *reciprocity (recip)* indicates the likelihood to reciprocate communication nominations (a tie from *i* to *j* is matched by a tie from *j* to *i*).
- *geometrically weighted edgewise shared partners* (*gwesp transitivity*) represents the tendency to create transitively closed triads [31, 32]. *gwesp transitivity* was utilized instead of *transitive triplets*, following recommendations in the literature [25, 30]. *gwesp transitivity* is similar to transitive triplets but considers additional transitive ties between the focal ego and alter scholars. Specifically, when *i* nominated *h* as a communication partner and *h* nominated *j* as a communication partner (*i to h to j*), gwesp transitivity indicates the likelihood of *i* nominating *j* as a communication partner. Additionally, gwesp transitivity introduces a scaling parameter *α* (0≤*α*≤∞) to account for the impact of additional transitive ties on the triad closure. For example, when an additional scholar, say *k*, was nominated by *i* and nominated *j*, the probability of *i* nominating *j* would be different from when there was one transitive tie (*i to h to j*). A scaling parameter *α* indicates that the additional transitive tie’s impact decreases, and the smaller the *α*, the less impact an additional transitive tie can offer [31]. We adopted the recommendation of *α* = *log*(2) = 0.69 [31], which resulted in a good model fit in our analysis. The interpretation of gwesp transitivity needs special care. For example, a positive gwesp transitivity indicates the log-odds of *i* nominating *j* as a communication partner if there were at least one additional scholar *h* who had been nominated by *i* and nominated *j*. The total log-odds of *i* nominating *j* would increase monotonically but non-linearly as the number of transitive ties increases when *α* = 0.69; this means that the increment of the log-odds by one increase of transitive ties decreases as the number of transitive ties increases. In other words, a positive gwesp effect implies excessive transitive triangle patterns among scholars, where transitive ties contribute to forming other triads with a marginally decreasing return to additional transitive ties [31].
- *three-cycles (cycle3)* indicate the propensity for closure in three-scholar communications (a tie from *i* to *j* and a tie from *j* to *h* predict a tie from *h* to *i*). *three-cycles* represent a non-hierarchical relationship pattern because the communication network among three scholars is cyclic (*i* to *h* to *j* to *i*, see the figure in S1 Table for a visualization of this relationship).
- *transitive reciprocated triplets* (*transRecTrip*) indicate the tendency of reciprocated communications between two scholars co-occurring with other communications through another scholar (a reciprocal communication tie between *i* and *j* if there are communications between *i* and *h* and *h* and *j*). Transitive reciprocated triplets are included to deal with spurious three-cycle effects, as suggested by Block [33].
- *in-degree popularity (inPopSqrt)* is the propensity for scholars with many incoming nominations to attract future communication nominations.
- *out-degree popularity (outPopSqrt)* is the tendency for scholars who name many others to attract nominations and *out-degree activity (outActSqrt)* is the inclination that scholars with many outgoing ties tend to send more ties at future time points. Square root transformation of these effects is applied to reflect the decreasing rates of the marginal growth of popularity and activity.
- *reciprocal degree-related activity* (*recipAct*) indicates an interaction between outdegree and reciprocity.
- *out-degree up to one* (*outTrunc(1)*) indicates a difference in the likelihood of communication between scholars with at least one outdegree and scholars with zero outdegree. A negative coefficient can be interpreted as a positive tendency towards outdegrees equal to 0 or being an isolate with respect to outgoing ties.

The perception change submodel includes the rate parameters to control for the volume of the changes during the two periods, as aforementioned. In addition, *linear* (*linear*) and *quadratic* (*quad*) shape parameters are included to capture the shape of the perception change. A positive linear shape effect indicates that there is a steady, linear increase in the perception of psychological safety over time. The inclusion of a quadratic shape effect allows for non-linearity of the change. A negative quadratic shape effect implies self-correction, while a positive one indicates addictive behaviors [19]. Linear shape and quadratic shape parameters are centered by subtracting the mean value of the target behavior (in this case, team psychological safety), which is a standard treatment in SAOM analysis.

*Model 2*: *Selection and peer influence model*. Model 2 tests the social selection and influence hypotheses. We included the *team psychological safety on rates* (*rate-TPS*) and the *team psychological safety similarity (sim TPS)* effects in the network change submodel. *rate-TPS* was used to test whether the expected number of opportunities for network changes was influenced by the scholars’ team psychological safety levels. A positive and significant effect would indicate that scholars with higher levels of team psychological safety had more opportunities to change their conversation ties. *Sim TPS* was used to test whether scholars tended to communicate with other scholars who were similar to themselves in terms of the team psychological safety levels. Similarity was measured for a given dyad as the absolute difference between the scholars’ team psychological safety scores, which was then reversely coded and centered using the average similarity across all possible dyads [25]. Higher values indicate greater similarity in the team psychological safety between the dyads. Thus, a positive and significant effect for psychological safety similarity indicates that two scholars who were similar in levels of team psychological safety had a higher likelihood of having conversations with each other, i.e., the presence of homophilous selection effects.

In addition, *average similarity in team psychological safety (avSim TPS)* was added to the perception change submodel to capture potential peer influence effects. The *average similarity* in team psychological safety is computed as the sum of the absolute differences in the psychological safety scores between egos and all other alters nominated by the egos. The sum was then reverse-coded, divided by the number of alters, and then centered based on the average level of similarity across all dyads [25]. A positive and significant effect of the average similarity suggests that scholars’ levels of team psychological safety became more similar to the peer scholars’ levels whom they conversed with during the mHTI program.

*Model 3*: *Full network submodel with background covariates*. Model 3 is designed to control for several other selection processes that could also promote conversations among the scholars with similar individual attributes, i.e., homophily. Diversity in demographic variables, such as race, gender, discipline, and cultural backgrounds could impact team collaboration dynamics [34]. Gender homophily is commonly observed in various social networks including communication networks [35–37]. Scholars’ disciplinary training as well as their team membership are expected to impact their communication networks. Thus, we considered gender, discipline, and team homophily in the network change submodel. Of note, age difference between team members could also play a role in explaining the scholars’ communication structures [34, 38, 39]. Since age information was unavailable in the data set, we considered scholars’ career stages as a proxy for age. However, most mHTI scholars were early- or mid-career professionals; i.e., they were similar in terms of career stages. Accordingly, career stage did not show significant effects in our analysis. Therefore, we proceeded with our analysis without including scholars’ career stage in the model.

An additional note is that we omitted *rate-TPS* and *outTrunc(1)* in Model 3 for the 2019 data set because 1) these two parameters were not statistically significant in Model 2 in that year, and 2) dropping the two parameters in Model 3 greatly improved convergence and model fit.

*Model 4*: *Full model with an alternative perception model specification on influence effects*. In Model 4, the hypothesis about peer influence is investigated with an alternative, more sophisticated perception model specification. Specifically, instead of *avSim TPS*, we considered the *average attraction toward lower psychological safety*(*avAttLower*) and the *average similarity among reciprocated ties* (*avSimRecip*) in the perception submodel. First, *avAttLower* can be understood as the influence of alter scholars with a lower level of team psychological safety than ego scholars. Technically, the *avAttLower* term makes the average similarity statistic only be influenced by the similarity scores of alters with lower team psychological safety levels (by setting the similarity scores of alters with higher team psychological safety levels to 1). The more similar the egos and alters, the closer the statistic gets to 1. A positive and significant effect of *avAttLower* indicates that ego scholars were more likely to decrease team psychological safety levels, i.e., there was an attraction towards lower psychological safety, when egos communicated with the scholars with lower team psychological safety levels. The attraction towards lower psychological would be stronger if alter scholars increased their psychological safety levels to match the egos’ safety levels. A negative and significant effect of *simAttLower*, on the other hand, indicates that ego scholars were less likely to decrease their psychological safety levels, i.e., there was a resistance towards lower psychological safety, when egos communicated with the scholars with lower team psychological safety. The resistance would be higher if alter scholars increased their team psychological safety to match the egos’ psychological safety levels. Second, *avSimRecip* can be understood as the influence of the alter scholars who had *reciprocal* conversations with the ego scholars. A positive and significant effect of *avSimRecip* suggests that the scholars’ team psychological safety levels became more similar among the scholars who had reciprocated communications with each other.

## Results

### Descriptive results

Table 2 summarizes the scholars’ background characteristics and temporal changes in their perceptions of team psychological safety. Between 5 and 7 scholars were assigned to each of the five teams per year. The mHTI organizers tried to maximize the diversity of each team by evenly allocating scholars with different disciplinary backgrounds across teams. With some exceptions, the attempts were generally successful as shown in Table 2. For example, in 2019 the gender proportion was somewhat uneven, and in 2017, some disciplines such as Computer Science and Medicine showed larger proportions than other disciplines. Table 2 also shows that the mHTI scholars’ perceptions of team psychological safety gradually increased over the course of the mHTI programs.

**Table 2:** Scholar’s background and TPS characteristics.

		2017		2018			2019	
**Background**		Freq. (%)		Freq. (%)			Freq. (%)	
Team	1	7 (20.0)		6 (20.7)			6 (20.7)	
	2	7 (20.0)		6 (20.7)			6 (20.7)	
	3	7 (20.0)		6 (20.7)			5 (17.2)	
	4	7 (20.0)		5 (17.2)			7 (24.1)	
	5	7 (20.0)		6 (20.7)			5 (17.2)	
Gender	Female	18 (51.4)		13 (44.8)			18 (62.1)	
	Male	16 (45.7)		16 (55.2)			11 (38.0)	
	Declined to state	1 (2.8)		0 (0.0)			0 (0.0)	
Discipline^1^	CS	13 (37.1)		5 (17.2)			8 (27.6)	
	MED	10 (28.6)		11 (37.9)			8 (27.6)	
	PSY	6 (17.1)		8 (27.6)			7 (24.1)	
	OTH	6 (17.1)		5 (17.2)			6 (20.7)	
**Target Variable**		Mean (SD)	α^2^		Mean (SD)	α^2^	Mean (SD)	α^2^
Team Psychological Safety	Day 1	4.85 (2.19)	0.81		4.63 (2.13)	0.69	4.65 (2.10)	0.77
	Day 3	5.35 (2.04)	0.82		4.48 (2.29)	0.72	5.00 (2.08)	0.78
	Day 5	5.65 (1.87)	0.80		5.39 (2.27)	0.88	5.42 (2.12)	0.85

^1^ Computer Science / Engineering / Data Science; MED: Medicine / Nursing; PSY: Psychology; OTH: Public Health / Others.

^2^ Cronbach’s α

Table 3 describes the characteristics of the scholars’ communication networks each year. Overall, scholars became more active in their conversations with other scholars as the training institute proceeded. There were generally a greater number of transitive triplets than non-transitive triplets in all three years, indicating that scholars’ triads were likely to be hierarchical in the observed data [40], meaning that if scholar *i* talked to scholar *j* and scholar *j* talked to scholar *h*, scholar *i* was also likely to talk to scholar *h* (but it was unlikely that scholar *h* talked to scholar *i*). Jaccard indices for each year are also shown in Table 3. The Jaccard index measures the stability of two successive networks. It is defined as the proportion of the number of ties occurring in both waves to the number of ties occurring in at least one wave [18, 25]. The Jaccard indices were over 0.5 in all three years, meaning that more than half of the conversation ties were preserved in successive networks.

**Table 3:** Characteristics of mHTI scholars’ conversation networks in 2017, 2018, and 2019.

	Day 1	Day 3		Day 5
	Value (%)	Value (%)		Value (%)
**2017**				
Ties	168 (14.1)	226 (19.0)		204 (17.1)
Outdegree	4.80	6.46		5.83
recip	44	50		53
cycle3	97	144		120
transTrip	396	612		577
Sim TPS	0.66	0.70		0.74
Same Team	132 (78.6)	128 (56.6)		133 (65.2)
Same GEN	79 (47.0)	111 (49.1)		88 (43.1)
Same DSC	38 (22.6)	65 (28.8)		57 (27.9)
**2018**				
Ties	119 (14.7)	178 (21.9)		178 (21.9)
Outdegree	4.10	6.14		6.14
recip	39	65		53
cycle3	81	145		130
transTrip	293	570		530
Sim TPS	0.65	0.63		0.67
Same Team	31 (26.1)	46 (25.8)		43 (24.2)
Same GEN	58 (48.7)	77 (43.3)		83 (46.6)
Same DSC	22 (18.5)	40 (22.5)		40 (22.5)
**2019**				
Ties	161 (19.8)	156 (19.2)		172 (21.1)
Outdegree	5.55	5.38		5.93
recip	58	64		56
cycle3	135	141		133
transTrip	495	510		537
Sim TPS	0.67	0.68		0.70
Same Team	125 (77.6)	130 (83.3)		119 (69.2)
Same GEN	75 (46.6)	83 (53.2)		93 (54.1)
Same DSC	28 (17.4)	30 (19.2)		35 (20.4)
**Jaccard index**	Day 1 to Day 3		Day 3 to Day 5	
2017	0.510		0.547	
2018	0.571		0.656	
2019	0.714		0.648	

Note. GEN is gender, and DSC is discipline. *Network Structure*. Ties is the number of ties in the network. *Outdegree* is the mean of scholars’ outdegrees. *Recip* is the number of reciprocal dyads. *Cycle3* is the number of non-transitive triplets (*i* to *h* to *j* to *i*). *TransTrip* is the number of transitive triplets (*i* to *h* to *j* and *i* to *j*). *Similarity* is the mean of similarity scores of team psychological safety between scholars. *Same* is the number of ties within the same group indicators. The Jaccard index measures the stability of two successive networks.

In addition, we measured the similarity of the scholars in terms of the target variable, scholars’ perceptions of team psychological safety. The similarity score of a scholar pair (*i*,*j*) is defined as 1−|*X*_*i*_−*X*_*j*_|/*Range*(*X*), where *Range*(*X*) is the range of covariate *X* (i.e., a maximum minus minimum of *X*) [25], where *X* is the levels of team psychological safety. By definition, similarity scores range from 0 (minimum) to 1 (maximum), and a larger score indicates greater similarity between two scholars in terms of the perception of team psychological safety. Table 3 lists the average similarity score over all the scholar pairs per day each year; the number of scholar pairs is *N*(*N*−1)/2, where *N* is the number of mHTI scholars each year. Overall, we observe an increasing trend in average similarity during the mHTI in all three years: as the institute proceeded, scholars appear to have become more similar to one another in terms of their perceptions of team psychological safety. We also measured the number of edges among scholars of the same gender, discipline, and team (i.e., homophily). The number of same-team edges was generally larger than the number of same-gender or same-discipline edges, except for 2018 that had fewer same-team and same-gender ties than other years.

**Fig 1:**
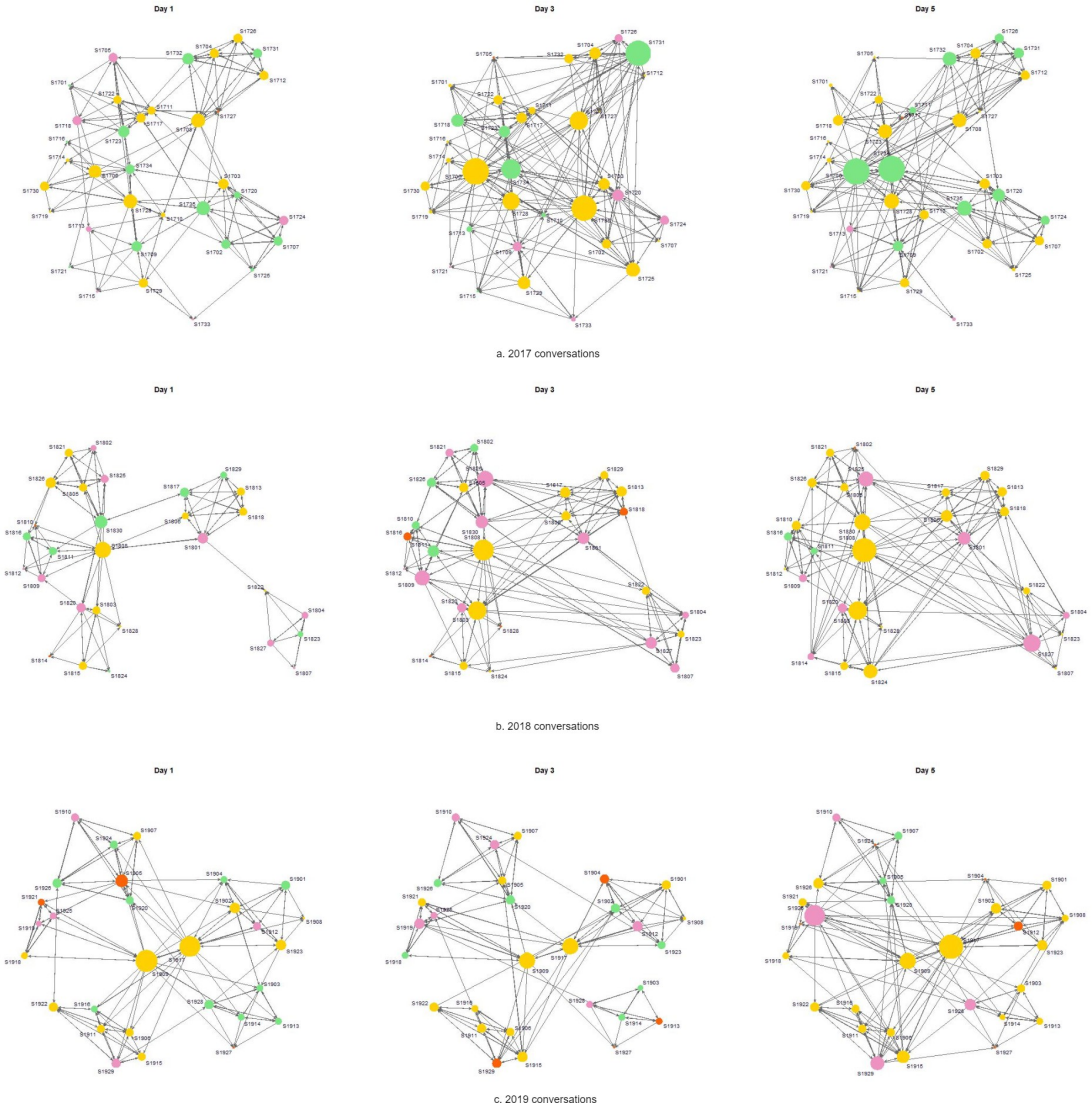
Scholars’ conversation network graphs with psychological safety homophily. Pink represents low scores, green represents medium scores, yellow represents high scores, and red represents missing scores (NA).

Fig 1 displays the scholars’ communication networks with psychological safety homophily. Here we classified the scholars into three groups based on their psychological safety levels: low (score = 0, 1, 2, 3), medium (score = 4, 5), and high (score = 6, 7), and they are shaded in different node colors in the graphs. Scholars who had conversations with each other are connected with solid arrows, and scholars with higher outdegrees are represented with larger node sizes. In Fig 1, we observe that on Day 1 there were a small number of scholars with a high level of team psychological safety, and they tended to initiate more conversations compared to others. On Days 3 and 5, more scholars showed medium and high levels of team psychological safety, and there were more active interactions among the scholars with all levels of team psychological safety. All other network visualizations with different homophily types are presented in the S1 Appendix.

### Model analysis results

We applied the four models described earlier to the three years’ data. Table 4 shows that all models converged adequately. The goodness-of-fit of the three years’ models was also satisfactory as shown in the graphs provided in the S2 Appendix. Table 5 lists the parameter estimates of the three models for each year. Statistical significance was presented with asterisks at the 0.001, 0.01, and 0.05 levels.

**Table 4:** Model convergence statistics for three years.

**2017**	Model 1	Model 2	Model 3	Model 4
convergence *t* ratios all	<0.09	<0.09	<0.10	<0.08
Overall maximum convergence ratio	0.17	0.21	0.23	0.24
**2018**	Model 1	Model 2	Model 3	Model 4
convergence *t* ratios all	<0.06	<0.08	<0.07	<0.08
Overall maximum convergence ratio	0.21	0.20	0.19	0.17
**2019**	Model 1	Model 2	Model 3	Model 4
convergence *t* ratios all	<0.13	<0.12	<0.09	<0.06
Overall maximum convergence ratio	0.18	0.21	0.22	0.19

Note. Overall, a maximum convergence ratio below the threshold of 0.25 and the t-ratio below the criterion of 0.10 indicate a model of interest converges.

**Table 5:** Parameter estimates of four SAOM models in 2017, 2018, and 2019.

	2017								2018								2019							
Effect	Model 1		Model 2		Model 3		Model 4		Model 1		Model 2		Model 3		Model 4		Model 1		Model2		Model 3		Model 4	
*Network Dynamics*																								
rate1	10.07	(1.75) ***	2.95	(1.34) *	4.43	(3.01)	4.44	(3.04)	8.07	(1.57) ***	12.71	(4.79) **	12.92	(4.83) **	12.45	(4.48) **	5.20	(1.46) ***	14.33	(9.46)	5.94	(1.68) ***	5.83	(1.67) ***
rate2	7.41	(0.91) ***	2.14	(0.97) *	2.38	(1.70)	2.38	(1.37)	5.84	(0.94) ***	9.19	(3.25) **	9.30	(2.90) **	9.01	(3.10) **	11.3	(3.91) **	29.48	(18.89)	14.59	(5.87) *	14.16	(6.68) *
rate-TPS			0.22	(0.08) **	0.25	(0.09) **	0.25	(0.11) *			-0.10	(0.07)	-0.10	(0.07)	-0.09	(0.06)			-0.22	(0.13)				
density	-0.63	(0.66)	-0.51	(0.59)	-2.89	(0.66) ***	-2.89	(0.69) ***	-0.95	(0.81)	-0.83	(0.84)	-0.87	(0.92)	-0.85	(0.83)	-0.91	(1.15)	-0.80	(1.15)	-4.59	(1.56) **	-4.75	(1.64) **
recip	2.15	(0.42) ***	2.11	(0.40) ***	0.97	(0.36) **	0.97	(0.38) *	3.20	(0.54) ***	3.15	(0.65) ***	3.12	(0.57) ***	3.15	(0.58) ***	2.33	(0.59) ***	2.44	(0.64) ***	0.66	(0.76)	0.64	(0.84)
transRecip	0.05	(0.13)	0.04	(0.13)	-0.06	(0.12)	-0.06	(0.13)	0.21	(0.13)	0.23	(0.14)	0.21	(0.14)	0.21	(0.15)	0.24	(0.16)	0.27	(0.19)	-0.09	(0.28)	-0.08	(0.28)
cycle3	-0.05	(0.13)	-0.05	(0.17)	-0.10	(0.13)	-0.10	(0.12)	-0.19	(0.15)	-0.21	(0.16)	-0.19	(0.16)	-0.20	(0.16)	-0.15	(0.22)	-0.15	(0.24)	0.04	(0.26)	0.03	(0.27)
gwesp transitivity	1.39	(0.23) ***	1.37	(0.24) ***	0.57	(0.29) *	0.57	(0.28) *	1.23	(0.27) ***	1.22	(0.25) ***	1.28	(0.27) ***	1.27	(0.28) ***	1.52	(0.36) ***	1.53	(0.35) ***	1.34	(0.41) **	1.32	(0.50) **
inPopSqrt	-0.84	(0.21) ***	-0.86	(0.21) ***	-0.30	(0.20)	-0.30	(0.21)	-1.04	(0.28) ***	-1.03	(0.28) ***	-1.06	(0.28) ***	-1.07	(0.29) ***	-0.89	(0.42) *	-0.96	(0.47) *	-0.61	(0.49)	-0.58	(0.45)
outPopSqrt	-0.26	(0.11) *	-0.26	(0.11) *	-0.07	(0.09)	-0.07	(0.09)	-0.16	(0.10)	-0.15	(0.10)	-0.15	(0.11)	-0.15	(0.11)	-0.44	(0.17) **	-0.51	(0.17) **	-0.62	(0.30) *	-0.59	(0.32)
outActSqrt	0.27	(0.13) *	0.25	(0.11) *	0.44	(0.13) ***	0.44	(0.12) ***	0.64	(0.23) **	0.60	(0.24) *	0.60	(0.24) *	0.60	(0.22) **	0.40	(0.22)	0.44	(0.22) *	0.60	(0.24) *	0.61	(0.31)
recipAct	-0.09	(0.06)	-0.08	(0.05)	0.01	(0.05)	0.01	(0.05)	-0.23	(0.08) **	-0.23	(0.09) **	-0.22	(0.08) **	-0.23	(0.07) **	-0.13	(0.09)	-0.15	(0.09)	0.22	(0.17)	0.22	(0.19)
outTrunc(1)	-1.69	(0.72) *	-1.79	(0.64) **	-2.13	(0.72) **	-2.14	(0.85) *	-2.97	(0.91) **	-2.98	(0.97) **	-2.96	(0.99) **	-3.00	(0.97) **	-2.95	(3.40)	-2.31	(2.50)				
same Team					1.86	(0.28) ***	1.85	(0.30) ***					0.35	(0.20)	0.36	(0.19)					3.15	(1.08) **	3.16	(0.93) ***
same GEN					-0.07	(0.12)	-0.07	(0.12)					-0.16	(0.16)	-0.16	(0.15)					0.88	(0.23) ***	0.88	(0.23) ***
same DSC					0.29	(0.13) *	0.29	(0.14) *					0.17	(0.18)	0.18	(0.18)					0.29	(0.23)	0.30	(0.30)
sim TPS			0.32	(0.39)	0.14	(0.39)	0.13	(0.51)			-0.48	(0.43)	-0.45	(0.44)	-0.47	(0.44)			-1.14	(0.54) *	-1.95	(0.86) *	-1.95	(0.76) *
*Target Psychological Dynamics*: *Team psychological safety *																								
rate 1	9.62	(9.32)	9.45	(7.71)	9.44	(8.31)	9.59	(4.15) *	3.72	(1.40) **	3.7	(1.29) **	3.75	(1.17) **	3.18	(0.98) **	4.07	(1.67) *	3.92	(1.39) **	3.91	(1.73) *	3.79	(1.25) **
rate 2	5.51	(3.00)	5.48	(2.24) *	5.39	(2.85)	5.58	(3.13)	5.63	(2.42) *	5.85	(3.02)	5.97	(2.70) *	5.44	(2.09) **	6.76	(8.27)	6.66	(6.18)	6.47	(3.21) *	6.23	(3.78)
linear	0.48	(0.11)***	0.47	(0.12) ***	0.48	(0.15) **	0.64	(0.11) ***	0.68	(0.20) ***	0.73	(0.18) ***	0.72	(0.21) ***	1.87	(0.28) ***	0.38	(0.13) **	0.34	(0.11) **	0.36	(0.11) **	-0.31	(0.13) *
quad	0.06	(0.03) *	0.07	(0.04)	0.07	(0.05)	0.09	(0.03) *	0.12	(0.04) **	0.08	(0.07)	0.07	(0.08)	0.08	(0.11)	0.07	(0.04)	0.15	(0.07) *	0.16	(0.08) *	0.13	(0.07)
avSim			0.54	(2.81)	0.33	(3.68)					-2.93	(4.77)	-2.86	(4.34)					4.91	(3.77)	5.02	(3.65)		
avAttLower TPS							3.41	(0.83)***							17.27	(0.61) ***							-10.09	(0.73) ***
avSimRecip							0.81	(2.27)							-11.10	(6.85)							8.36	(3.09) **

Notes. Significance levels.

*** indicates *p*<0.001

** indicates *p*<0.01

* indicates *p*<0.05. Network Dynamics. *rate1* (and *rate2*) is the expected constant rate of the network change in period 1 (and 2): day 1 to 3 (and day 3 to 5). *density* is the degree of network ties (out-degree). *recip* represents reciprocal ties. *transTrip* represents transitive triads. *cycle3* is triad closure with no reciprocated ties. *transRecTrip* is the reciprocated ties in the transitive triad. *inPopSqrt* is in-degree popularity in square root form. *outPopSqrt* is out-degree popularity in square root form. *outActSqrt* is out-degree activity in square root form. *recipAct* is the reciprocated ties activity. *sim TPS* is the similarity score of team psychological safety between scholars. *same* is the homophily effect of a given group.

Target Psychological Dynamics. *rate1* (and *rate2*) is the expected constant rate of the perception change in period 1 (and 2). *linear* is linear shape. *quad* is quadratic shape. *avSim* TPS is the average similarity of team psychological safety. *indeg* is in-degree. *outdeg* is out-degree. *avAttLower* TPS is the one-sided version of avSim. *avSimRecip* is the influence from reciprocated ties.

### Model 1: Basic model

The parameter estimates of Model 1 are presented in the first sub-column of the 2017, 2018, and 2019 columns.

*Network change submodel* In all three years, positive and significant rate effects were observed, which indicate significant changes in communication ties throughout the mHTI. The following patterns were also commonly observed across years: the outdegree (*density*) parameter was not statistically significant, which means after controlling for network structural effects, the probability of a tie being created was not significantly different from zero, indicating that scholars did not appear to be selective in their communication partner nominations. Reciprocity (*recip*) was significant and positive, indicating that scholars tended to reciprocate communication nominations. Transitive reciprocated triplets (*transRecTrip*) and three-cycle effects (*cycle3*) were not statistically significant in all three years. However, we found positive and significant effects for geometrically weighted edgewise shared partners (gwesp transitivity), which implies that ego scholars were more likely to talk to an alter scholar who was talked to by a scholar with whom the ego had already communicated. The probability of scholars’ talking to other scholars in this form would increase as the number of transitive ties increased, while the transitive ties’ contributions to forming other triads would decrease marginally.

The in-degree popularity (*inPopSqrt*) and out-degree popularity (*outPopSqrt*) were negative and significant in all three years, indicating that the scholars were less likely to have conversations with other scholars with many in-coming or out-going communications. Out-degree activity (*outActSqrt*) was positive, indicating that those scholars with many out-going communications were more likely to generate even more communications over time. Reciprocal degree-related activity effects (*recipAct*) were negative and significant in 2018, implying that among scholars with the same number of reciprocal ties, the scholars with more outdegrees were less likely to generate communications than others with fewer outdegrees. Zero-degree effect (*outTrunc(1)*) was negative and significant in 2017 and 2018, suggesting there were positive tendencies for the scholars not to initiate new communications during the mHTI in those two years.

*Perception change submodel* The two rate parameters (*rate1* and *rate2*) were positive in all three years, but some parameters were not statistically significant (*rate1* and *rate 2* in 2017 and *rate2* in 2019). The significant and positive rates indicate that scholars experienced successive and significant changes in the perception of psychological safety over the course of the mHTI training week. The linear shape parameters were positive and significant in all three years and quadratic (*quad*) shape parameters were positive and significant in 2017 and 2018, indicating that the scholars’ perceptions of team psychological safety tended to increase during the mHTI training program in a linearly increasing and then slightly accelerating pattern in those years.

### Model 2: Selection and influence effects

The parameter estimates of Model 2 are presented in the second sub-column of the 2017, 2018, and 2019 columns. The parameter estimates included in Model 1 remained similar unless otherwise mentioned. Hence, here we focus on the parameters newly added to Model 2.

*Network change submodel* The selection effect, represented by the perception similarity parameter (*sim TPS*), showed somewhat different patterns across three years. The effect was positive but statistically not significant in 2017, negative but not significant in 2018, and negative and significant in 2019. The negative and significant *simTPS* indicates that scholars in 2019 with similar levels of psychological safety were less likely to communicate with one other. We tested whether the network change would be impacted by the levels of team psychological safety. The impact was positive and significant in 2017, indicating scholars with higher levels of team psychological safety would expect more opportunities to make a change in their project-based communications. We also tested the effects of ego’s and alter’s psychological safety levels on network change. The ego/alter main effects were not statistically significant, and the estimates of the similarity effects did not change much, with and without the ego/alter main effects. We thus excluded the ego and alter main effects from the model. The rate parameters lost significance in 2019 when the above effects were added.

*Perception change submodel* The estimate of peer influence represented by average similarity in the psychological safety (*avSim TPS*) was not statistically significant in any of the three years. That is, the scholars’ perceptions of psychological safety did not appear to be influenced by the average psychological safety levels of the scholars with whom they spoke. We also tested the scholars’ indegree and outdegree effects on the change in levels of team psychological safety, but they were not statistically significant in any year. We thus excluded these effects from the model.

### Model 3: Controlling for scholars’ attributes

The parameter estimates of Model 3 are presented in the third sub-column of the 2017, 2018, and 2019 columns.

*Network change submodel* After controlling for scholars’ attributes, the density parameter was significant and negative in Model 3 in the 2017 and 2019 networks: hence, given the same levels of attributes, the scholars were unlikely to initiate communications with other scholars in the network. The reciprocity increased the log-odds of sending a tie by 0.97 in 2017, by 3.12 in 2018, and by 0.66 in 2019, all else being equal. *The gwesp transitivity* effects stayed positive and statistically significant in all three years, indicating that scholars were likely to nominate a scholar who was his or her communication partners’ communication partner, as a new communication partner. For example, in 2019, the log-odds of a scholar nominating another scholar as communication partner was 1.34 if there were only one transitive communication partner between them. Additional transitive communication partners would continue to increase the log-odds but at a decreasing rate; specifically, the second transitive communication increases the log-odds by 0.67, and the third one increases the log-odds by 0.34. The in-degree popularity lost its significance in 2017 and 2019, and out-degree popularity was only significant in 2019. For the 2019 network, Model 3 with zero-degree effects did not converge. After removing the effect of *TPS on rates* (*rate-TPS*), the model fit was improved. Therefore, we proceeded with Model 3 without these two effects (*zero-degree*, *rate-TPS*) for the 2019 network.

Team homophily (*same Team*) was positive and significant in the 2017 and 2019 networks. Shared team membership would increase the log-odds of sending a tie by 1.86 in 2017, by 0.35 in 2018, and 3.15 by 2019, all else being equal. In other words, scholars were more likely to have project-based conversations with their team members than scholars outside their teams, which is not surprising given that scholars were engaged in team-based projects during the mHTI. Gender homophily (*same GEN*) was positive and significant in 2019 (a within-gender tie is 1.41 times more likely to be created, all else being equal) and discipline homophily (*same DSC)* was positive and significant in 2017 (a within-discipline tie is 1.14 times more likely to be created, all else being equal), indicating that the role of scholars’ backgrounds in their communications was different across years. The mixed homophily effects across years might be explained by the differences in team compositions and collaboration dynamics among the scholars with similar backgrounds. For example, we found significant discipline homophily in 2017, when 37% of the participants came from computer science (CS). On the other hand, we found no discipline homophily in 2018 even though 38% of the participants came from the field of medicine. A possible explanation for these results could be that the computer science discipline in the mHealth context requires a relatively narrow spectrum of expertise, meaning that the computer science scholars were homogeneous in terms of their expertise, which led to more frequent discussions among themselves and accordingly, significant discipline homophily effects. On the other hand, the field of medicine may tap into a wider spectrum of specialized, disease-specific knowledge and expertise, which might have resulted in less frequent discussions among the participating physicians, and accordingly, no significant discipline homophily effects. We detected a negative and statistically significant effect of social selection on team psychological safety in 2019, indicating that the 2019 scholars were more likely to have conversations about the projects with those who had different levels of team psychological safety. Specifically, the log-odds of a tie being created between two scholars with an additional level of difference in psychological safety was 0.28. For example, sending a tie between scholars with 3-point difference in team psychological safety is 1.40 times more likely to occur than that between scholars with the same team psychological safety, all else being equal; {1/(1+*exp*(−0.28*3))}/{1/(1+*exp*(0))} = 1.40.

*Perception change submodel* Based on Model 2, we further added scholars’ background variables to the perception change submodels to account for the effects of scholars’ background characteristics on their change in psychological safety (not shown in Table 5). We tested the effects of individuals’ backgrounds, such as gender and discipline, but those effects were not statistically significant in the perception change submodels. Therefore, we removed these covariate effects from the model.

Of note, we tested for time heterogeneity [41] of the identified effects in all considered models, because team collaboration is a dynamic phenomenon and can have different foci at different times [42]. For example, an effect could be stronger during one transition period (Day 1 to Day 3) than at the other, later stage (Day 3 to Day 5). In this case, however, the model with time heterogeneity effects did not significantly improve the overall model fit, and in fact, it worsened the model convergence. Thus, we reported all results based on the models without time heterogeneity.

### Model 4: Taking a closer look at social influence

The model parameter estimates of Model 4 are presented in the last sub-column of the 2017, 2018, and 2019 columns.

*Network change submodel* The network change submodel for Model 4 stayed the same as Model 3, and the parameter estimates showed minimal changes in terms of statistical significance. The outdegree popularity and outdegree activity parameter lost significance in 2019. Otherwise the results stayed consistent between Model 3 and Model 4. In addition, we tested a four-parameter specification of actor-level covariates [43] in order to differentiate different mechanisms of homophily. However, the effects of the additional parameters from the four-parameter specification were not statistically significant. Therefore, we decided not to include those additional effects.

- *Perception change submodel* The main change made in Model 4 is that we replaced the social influence effect measured by average similarity of connected scholars’ team psychological safety in Model 3 with two separate parameters in Model 4: the average attraction towards lower team psychological safety effect (*avAttLower TPS*) and an interaction between average similarity and reciprocity (*avSimRecip*). Interestingly, the three years presented somewhat different patterns in terms of these two separate parameters. The positive and significant *avAttLower TPS* effect in 2017 and 2018 implies that there were social influences from the scholars with lower team psychological safety levels, and such effects would influence the scholars to decrease their psychological safety levels to become more similar to their peers. On the other hand, the *avAttLower TPS* effect was negative and significant in 2019. This indicates that scholars were influenced by other scholars who had lower team psychological safety levels than themselves; unlike 2017 and 2018, though, scholars would not lower their team psychological safety levels, especially when alter scholars increased their levels of team psychological safety to match those of the ego scholars. In addition, we found significant *avSimRecip* effects in 2019 only. The positive *avSimRecip* effect indicates that scholars in 2019 were positively influenced by the alter scholars with whom they had reciprocated communications. Specifically, if all reciprocated communication partners had higher psychological safety, the ego scholars would increase their psychological safety levels by 1 point with probability of 0.765.

## Discussion

With our analytic framework, we related the dynamics of scholars’ project-focused conversation networks to changes in their perceptions of team psychological safety during three annual mHTIs. Jointly modeling scholars’ communication networks and their perceptions of psychological safety helped us obtain nuanced insights about team collaboration dynamics during the mHTIs. Below we summarize our main findings.

First, in terms of change in the scholars’ communication network during the mHTI, the network generally grew larger during the training program. Specifically, there was an increase in the number of scholars who actively initiated communications with other scholars as the week-long training progressed. The growing number of reciprocated ties showed that reciprocity was an important mechanism in network expansion. Interestingly, the effects of reciprocated ties varied across years. For example, while reciprocal ties served as a channel to transport social influence in 2019, in 2018 the likelihood of the scholars with more reciprocal ties initiating new ties was diminished by their nominations (outdegrees) of other scholars. Along with the network expansion, popular scholars who had received more nominations from other scholars were less likely to receive a new nomination and also less likely to send nominations to other scholars. However, active scholars who had sent out more nominations to other scholars were more likely to initiate new conversations in all three years. In addition, we observed that scholars tended to communicate in transitive triads (*gwesp transitivity*) after controlling for scholars’ background attributes and similarities in those attributes. This pattern suggests that in an intensive scientific collaboration program like the mHTI, communicating with scholars who communicated with other scholars was likely to bring about direct communications between the scholars and the communication partners of their communication partners. These kinds of interactions could help accelerate the spreading of shared knowledge even when scholars might not be acquainted with all other scholars. For example, suppose scholar *i* had a question about software development and asked scholar *h* for help, and scholar *h* happened to talk to scholar *j* who was an expert in software engineering. A positive gwesp transitivity indicates that scholar *i* was likely to talk to scholar *j* given the transitive communication tie (*i* to *h* to *j*). More of such ties would further increase the likelihood of a direct communication from scholar *i* to *j*, and accordingly, knowledge transfer between them.

Second, the scholars’ perceptions of psychological safety changed during the training program, more consistently between Day 1 and Day 3, rather than between Day 3 and Day 5. After controlling for social influence through communications with scholars, we found linear trends in changes of team psychological safety during the institutes. Individual background attributes, such as gender and discipline, did not show statistically significant effects in the perception change submodels. Therefore, those covariates were not included in the final perception change submodel.

Our analysis showed interesting findings about peer influence. For example, peer influence was not statistically significant when considered as the average similarity of a scholar’s team psychological safety level to the levels of their conversational partners. However, scholars were more likely to be impacted by the scholars who had lower levels of team psychological safety, although their responses to those influences differed somewhat across years. For instance, in 2017 and 2018, scholars were likely to be influenced by peers who had lower psychological safety levels than themselves. However, in 2019, scholars were less likely to be influenced by those with lower psychological safety. In addition, in 2019, scholars’ team psychological safety levels were positively influenced by other scholars with whom they had reciprocated communications, whereas in 2017 and 2018, the influence from other scholars with reciprocated communications was not statistically significant.

We found evidence of selection effects, i.e., that similarity in perceptions of psychological safety did influence how the scholars selected their conversation partners during the mHTI. Specifically, in the 2019 data, mHTI scholars tended to interact more often with other scholars who had dissimilar perceptions of psychological safety. Such a phenomenon is often observed in early team building stages [44] as participants communicate to try to understand the viewpoints of team members—especially those who think differently than they do—in order to progress toward a shared team mentality. Thus, we view the identified selection effect as suggesting efforts to involve others with different levels of psychological safety in project-based conversations. The short duration of the week-long program might also have intensified the need to learn quickly about differing points of view within the team in order to produce a high-quality, team-based output efficiently.

We observed additional heterogeneity in the results across years. For example, team homophily was positive and significant in the 2017 and 2019 networks but was not significant in 2018. This is an interesting result because the mHealth program itself did not differ substantially in 2018, as compared to the 2017 and 2019 programs. Thus, we can only speculate why the 2018 data did not show significant team homophily, unlike in 2017 and 2019. The group dynamics of the 2018 cohort might have been different. For example, several scholars were senior faculty in their home institutions in 2018 and fewer within-team conversations were observed in that year. We speculate that those 2018 senior scholars might have been more extroverted and thus more inclined to converse with other scholars outside their teams, which might have resulted in lack of team homophily in the 2018 sample. As mentioned earlier, we observed that the social selection and social influence effects also varied in terms of sizes, directions, and statistical significance across the three years. This indicates that team psychological safety might be sensitive to the contexts of scholars’ collaborations, such as team composition and dynamics between team members, even though the content of the mHTI program was the same.

### Significance

We demonstrated the use of model-based longitudinal network analysis with SAOMs for examining the relationships between changing social interactions among participants and changing perceptions of team psychological safety and communication behaviors during an interdisciplinary training program. SAOMs have been widely used in social science research [19, 22, 45–49], but SAOMs and other longitudinal network analyses have rarely been applied to examine team science processes. Research findings on team science have primarily shown that team training improves team cognitive, affective, process, and performance outcomes [50]. Hence, growing interest has emerged in applying longitudinal network analysis to understand how teamwork and training processes deliver successful outcomes [e.g., 51]. However, most published work to date has been limited to descriptive analysis of longitudinal network data, rather than model-based analysis [e.g., 51–54].

Like any other generalized model based on observational data, SAOM analysis results do not imply causality [25]. SAOM analysis still enables us to take a deeper look at the process of human interactions over time, helping disentangle the two intertwined mechanisms, social selection and influence, in ways not possible with most static network analysis models. Specifically, our model-based dynamic network analysis approach enables team science researchers and evaluators to identify factors that could motivate or hinder effective team communications and collaborations. Our analysis of the mHTI data showed how factors, such as team membership, disciplinary background, and gender [55, 56], can explain the structure, formation, or changes of scholars’ communication networks. For example, scholars from the same team were more likely to communicate in 2017 and 2019, scholars of the same gender were more likely to communicate in 2019, and scholars of the same discipline were more likely to communicate in 2017. Importantly, our approach enabled us to test two potential mechanisms—social selection and peer influence—to explain how scholars’ perceptions of team psychological safety were influenced by the varying perceptions of the others with whom scholars spoke, and how the choice of conversation ties was influenced by the attributes of others. We identified evidence of social selection in terms of perceptions of psychological safety during the mHTI, which suggests that understanding the role of team members’ psychology can be useful to promote successful team communications and collaborations. Training institutes like the mHTI can use instruments such as the Team Psychological Safety Scale to monitor the selection mechanisms and intervene if necessary. In the 2019 data, we also found that scholars tended to converse more with scholars who showed a larger difference in their perception of team psychological safety (*negative simTPS*). This finding indicates that scholars with low team psychological safety levels were more likely to communicate with scholars who had higher psychological safety levels. This means that scholars with high team psychological safety levels were likely exposed to social influences from scholars with lower team psychological safety levels, although we found a resistance to such influences (*negative avAttLower*). Overall, these results suggest that it would be helpful if the mHTI program could include activities that could encourage more positive interactions among team members to further promote psychological safety within teams.

Taken together, the analytic framework that we employed in this study allows us to integrate and simultaneously examine factors involved in the formation of team science networks of interest, temporal changes in participants’ malleable behaviors or psychological perceptions, and the influences of participants’ unmalleable attributes. We have demonstrated in our study that the proposed framework is a promising methodology that has the potential to inform future SciTS research and evaluation. The dynamic perspectives taken in the framework can not only describe how the human networks and psychological traits of interest change over time. The analytic approach can also explain how the changes are derived, motivated, and influenced by each other, informing administrators and evaluators of team science programs on the process and components of successful team communications and collaborations.

The current study is not without limitations and there are avenues for further improvement. For example, the outcome variable, perception of team psychological safety, was measured by a 7-point Likert scale. If we used the raw item-level responses to create the test-level scores by summing them, the test-level scores would range from 7 to 49, which might be considered too large, potentially resulting in unstable estimation in RSiena. Thus, we dichotomized the item-level scores and then calculated the test-level sum scores, which range from 1 to 7. We believe such manipulation was a reasonable choice given the situation, but potentially useful information in the data might have been lost by doing so. In addition, the sample size of our empirical data might be small for identifying nodal attribute effects with SAOM with sufficient power. We considered combining the three years of network datasets (2017, 2018, and 2019) and analyzing one large network instead of three small networks. Doing that, however, introduces an assumption that the team collaboration process was homogeneous (i.e., following a universal mechanism across the years). To evaluate such an assumption, we conducted the heterogeneity test using a multiple-group SAOM model [25]. However, the homogeneity null hypothesis was rejected (chi-squared = 445.80, p<0.0001), suggesting that assuming a homogeneous collaboration process for three waves of network data would be undesirable for the data being analyzed. Therefore, we stayed with three separate network data analyses. In a future study, we hope to have the opportunity to apply the specified model to larger data sets to validate the results that we found in the current study.

## Supporting information

S1 Table Model specification of SAOM of a project-based network and perceptions of psychological safety. (DOCX)

S1 Appendix Network graphs over three years of the mHealth Institute. (DOCX)

S2 Appendix Goodness of fit plots. (DOCX)
