# Supplementary material for: Relationships between changing communication networks and changing perceptions of psychological safety in a team science setting: Analysis with actor-oriented social network models
Source: PLoS One. 2022 Aug 31;17(8):e0273899. doi: 10.1371/journal.pone.0273899 (PMC9432705; doi:10.1371/journal.pone.0273899)

**S1 Table. Model specification of SAOM of a project-based network and perceptions of psychological safety**

| Effects | Mathematical Formulation | Definition |
| --- | --- | --- |
| **Network Change Model** | | |
| Outdegree (density) | $\sum_{j} x_{ij}$ | number of out-going ties; measuring activity of the project-based conversations |
| Reciprocity (recip) | $\sum_{j} x_{ij}x_{ji}$ | the number of mutual ties; measuring the reciprocity of the network |
| Transitive triples (transTrip) | $\sum_{j,h} x_{ij}x_{ih}x_{hj}$ | the number of project-based conversations that occurred between *i* and *j* through a third scholar *h*, while *i* had talked to *j*; measures the tendency to network closure |
| Transitive reciprocated triplets (transRecTrip) | $\sum_{j,h} x_{ij}{x_{ji}x}_{ih}x_{hj}$ | The number of project-based conversations that occurred between *i* and *j* through a third scholar *h*, while *i* and *j* had mutually talked |
| Three cycles (cycle3) | $\sum_{j,h} x_{ij}x_{jh}x_{hi}$ | the number of cycled project-based conversations among three scholars |
| Geometrically weighted edgewise shared partners (GWESP(FF, $\alpha$)) | $\sum_{j=1} x_{ij}e^{\alpha}\{1-\left( 1-e^{-\alpha} \right)^{\Sigma_{h=1}x_{ih}x_{hj}}\}$ | The weighted number of transitive triplets between scholars *i*, *h*, and *j* where there are two paths from scholars *i* to *j* such that scholar *i* to *j* and scholar *i* to *h* to *j.* |
| In-degree related popularity sqrt effect (inPopSqrt) | $\sum_{j} x_{ij}\sqrt{\sum_{h} x_{hj}}$ | the sum of the square roots of the in-degrees of alters to whom *i* is tied; measures the attraction of the scholars due to their current popularity |
| Out-degree related popularity sqrt effect (outPopSqrt) | $\sum_{j} x_{ij}\sqrt{\sum_{h} x_{jh}}$ | the sum of the square roots of the out-degrees of alters to whom *i* is tied; measures the activity of scholars due to their current popularity |
| Out-degree related activity sqrt effect (outActSqrt) | $\sum_{j} x_{ij}\sqrt{\sum_{j} x_{ij}}$ | the cross-product of the scholars’ out-degrees and square root of out-degrees; measure the activity of scholars due their current activity |
| Reciprocal degree-related activity effect (recipAct) | $\left( \sum_{j} x_{ij} \right)*\left( \sum_{j} x_{ij}x_{ji} \right)$ | The outdegree of scholar *i* multiplied by scholar *i*’s reciprocity |
| Truncated out-degree up to *p* (outTrunc(*p*)) | $min(x_{i+}, p)$ | Outdegree truncated to *p* if a scholar’s outdegree exceeds *p*. In this study $p=1$, meaning that scholars who sent at least one tie have 1, but scholars who sent no ties have 0. |
| Covariate-related similarity (sim z) | $\sum_{j} x_{ij}(sim_{ij}^{Z}-\bar{sim^{Z}})$ | the sum of centered similarity scores of Z between scholars and the others to whom he is tied; measures the tendency of ego discussing projects with scholars who shared the a similar level of Z |
| Same covariate (same X) | $\sum_{j} x_{ij}I\{X_{i}=X_{j}\}$ | the number of ties that the ego sends to all other scholars who have exactly same value on X; measures the tendency of ego discussing projects with scholars who have the exact same values of X |
| **Behavior Change Model** | | |
| Linear shape effect (linear) | $z_{i}$ | the basic drive towards high levels of the behavior |
| Quadratic shape effect (quad) | $z_{i}^{2}$ | the effect of behavior on itself |
| average similarity effect (avSim) | $\sum_{j} x_{ij}(sim_{ij}^{Z}-\bar{sim^{Z}})/\sum_{j} x_{ij}$ | measures the preference of scholars to being similar with their alters in terms of Z |
| Average attraction toward lower effect (avAttLower) | $\left( \sum_{j} x_{ij} \right)^{-1}*\sum_{j} x_{ij}(1-\frac{max(z_{j}-z_{i},0)}{Range\left( X \right)})$ | The average similarity between scholar *i* and alter scholar *j*’s, where the similarity is replaced by 1 for those who have a higher behavior than *i*. |
| Average similarity between reciprocated ties (avSimRecip) | $\left( \sum_{j} x_{ij}x_{ji} \right)^{-1}*\sum_{j} x_{ij}x_{ji}(sim_{ij}^{Z}-\bar{sim^{Z}})$ | The average similarity of scholar *i* only with scholar *j*’s who have reciprocated ties with scholar *i* |

- $sim_{ij}^{X}=1-\frac{|X_{i}-X_{j}|}{Range(X)}$
- $I\{X_{i}=X_{j}\}$ is an indicator function, which takes the value of 1 when $X_{i}=X_{j}$, and 0 otherwise.

Below diagrams visualize what reciprocity, three cycles, transitive triples, transitive reciprocated triplets, and geometrically weighted edgewise shared partners mean in the network.


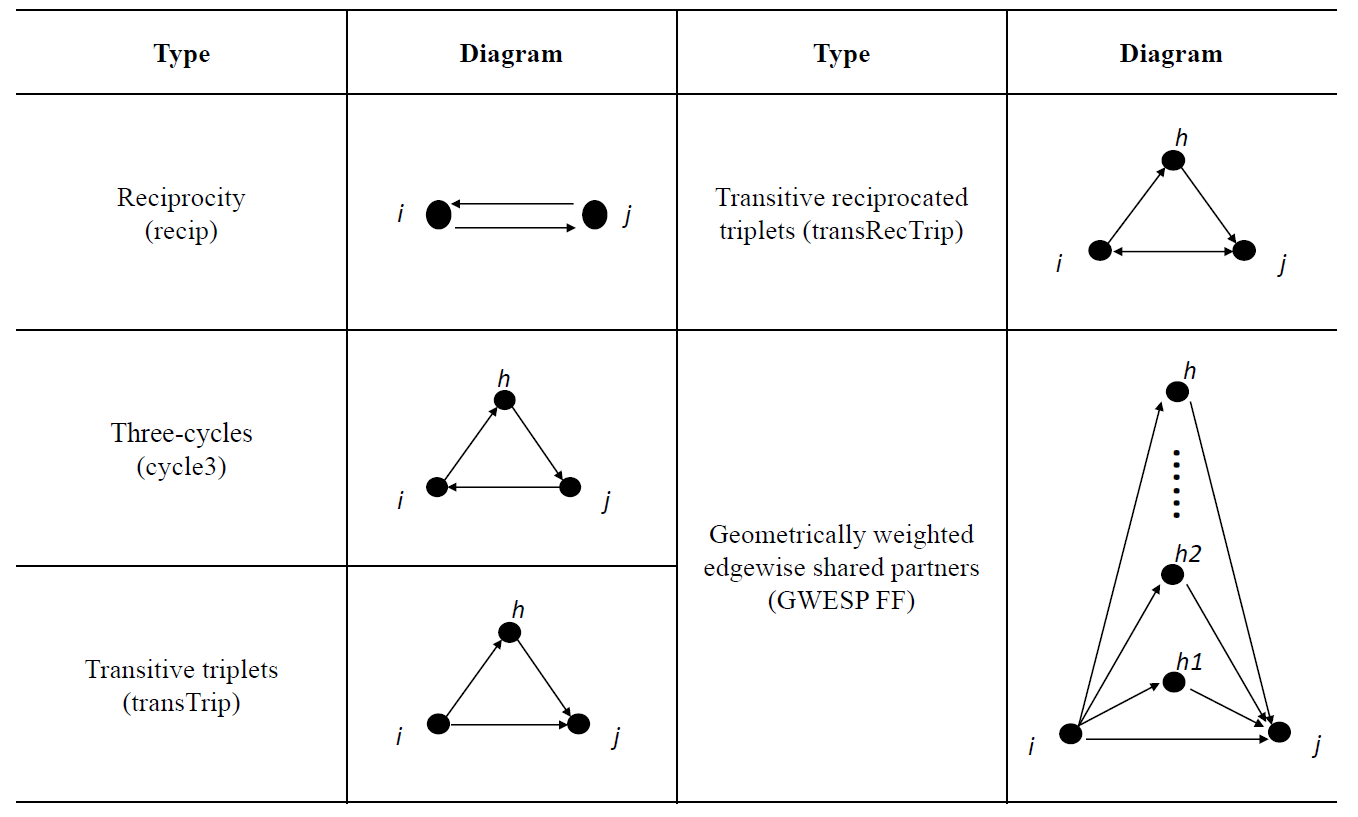

Supplement: S1 Table — (DOCX) [file pone.0273899.s001.docx]
