# Supplementary material for: Relationships between changing communication networks and changing perceptions of psychological safety in a team science setting: Analysis with actor-oriented social network models
Source: PLoS One. 2022 Aug 31;17(8):e0273899. doi: 10.1371/journal.pone.0273899 (PMC9432705; doi:10.1371/journal.pone.0273899)
Supplement: S1 Appendix — (DOCX) [file pone.0273899.s002.docx]

**S1 Appendix. Network graphs over three years of the mHealth Institute**

Network graphs for project based conversation over three years are presented. In network graphs, circles represent scholars, and the size of circles show relative magnitudes of scholars in network in terms of outdegree measure. Directed arrows represent the actual conversations between scholars (ties) during the institute. In order to show homophily and similarity between scholars, we use different colors for scholars (circles) based on scholar’s group membership as follows:

- Team membership: pink for team 1, green for team 2, yellow for team 3, red for team 4, skyblue for team 5.
- Gender: green for female scholars and red for male scholars.
- Discipline: pink for Computer Science/Engineering/Data Science, green for Medicine/Nursing, yellow for Psychology, and red for Public Health/Others.

| 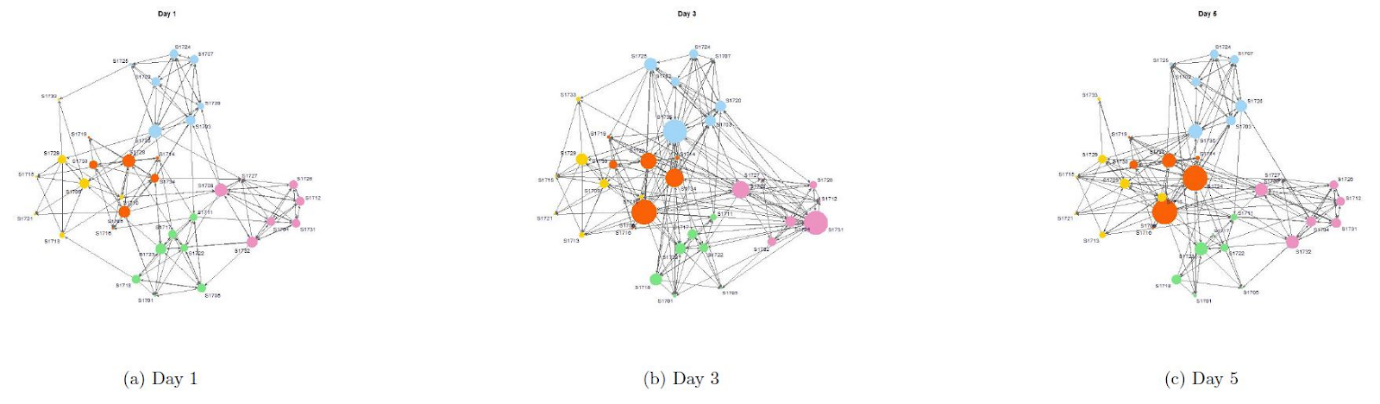  (a)Team: team 1 (pink), team 2 (green), team 3 (yellow), team 4 (red), and team 5 (sky blue) |
| --- |
| 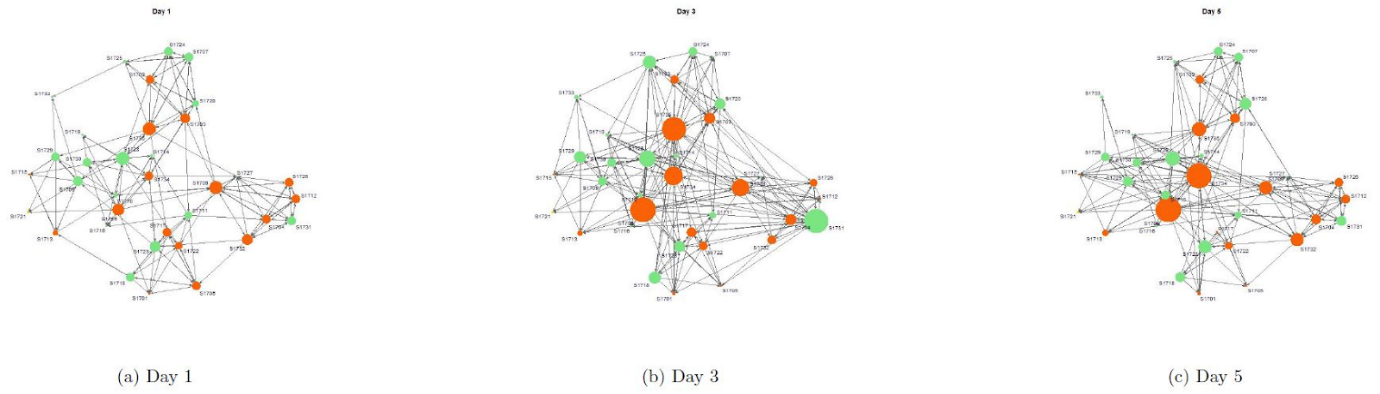  (b)Gender:  Female (green), Male (red), Decline to state (yellow) |
| 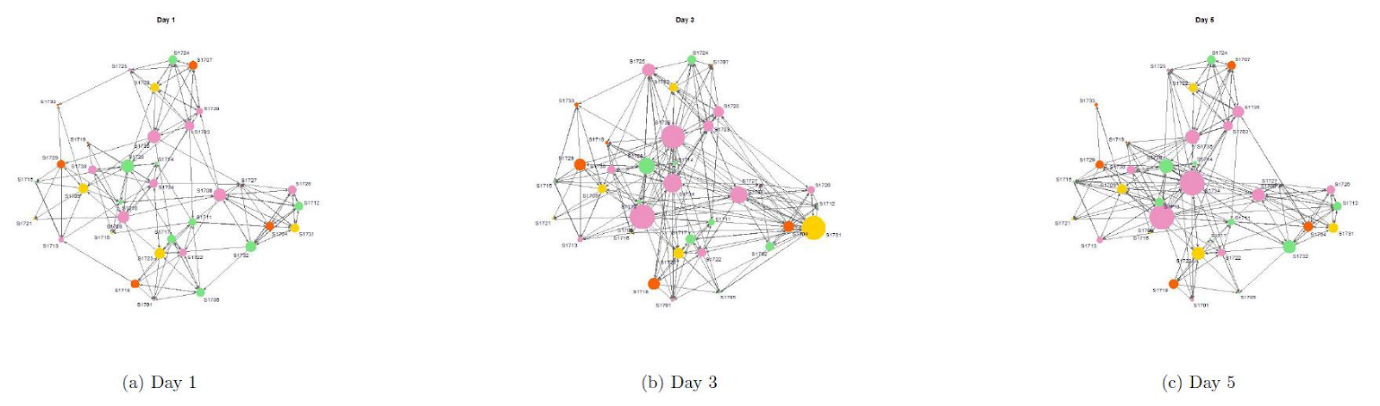  (c)Disciplines: Computer Science / Engineering / Data Science (pink), Medicine / Nursing (green), Psychology (yellow), and Public Health / Others (red) |

*S1-1 Fig. 2017 project based conversations by team(a), gender(b), and discipline(c)*

| 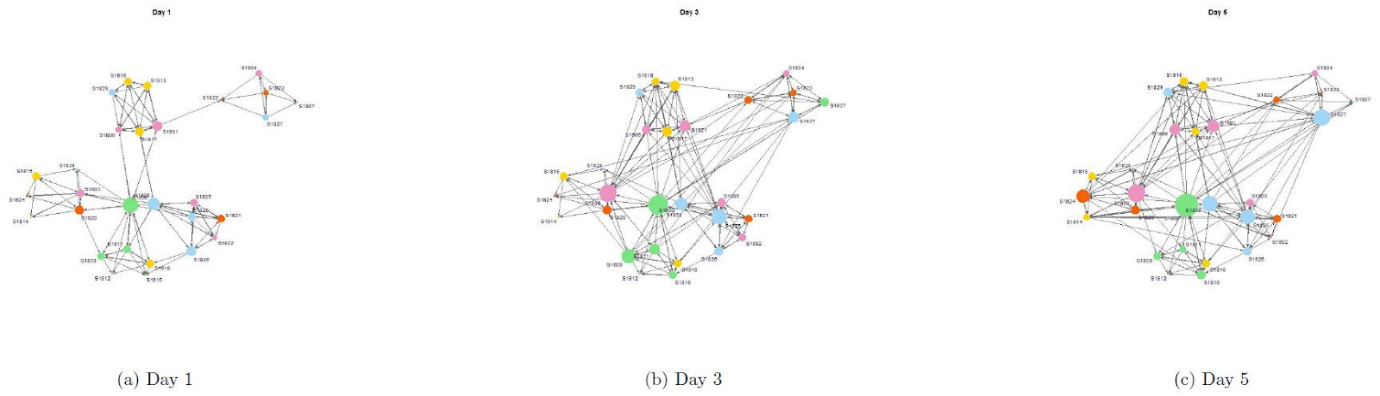  (a)Team: team 1 (pink), team 2 (green), team 3 (yellow), team 4 (red), and team 5 (sky blue) |
| --- |
| 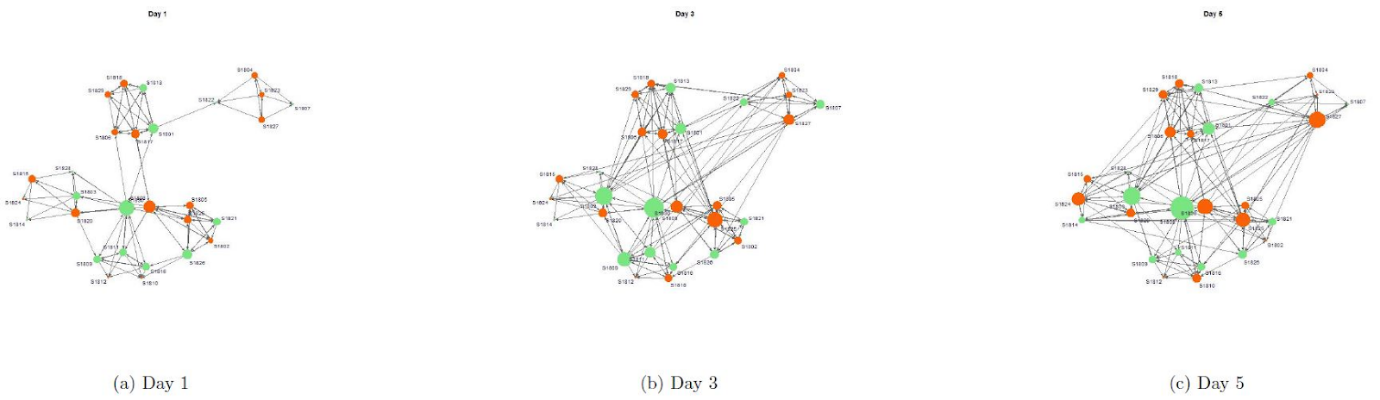  (b)Gender:  Female (green), Male (red), Decline to state (yellow) |
| 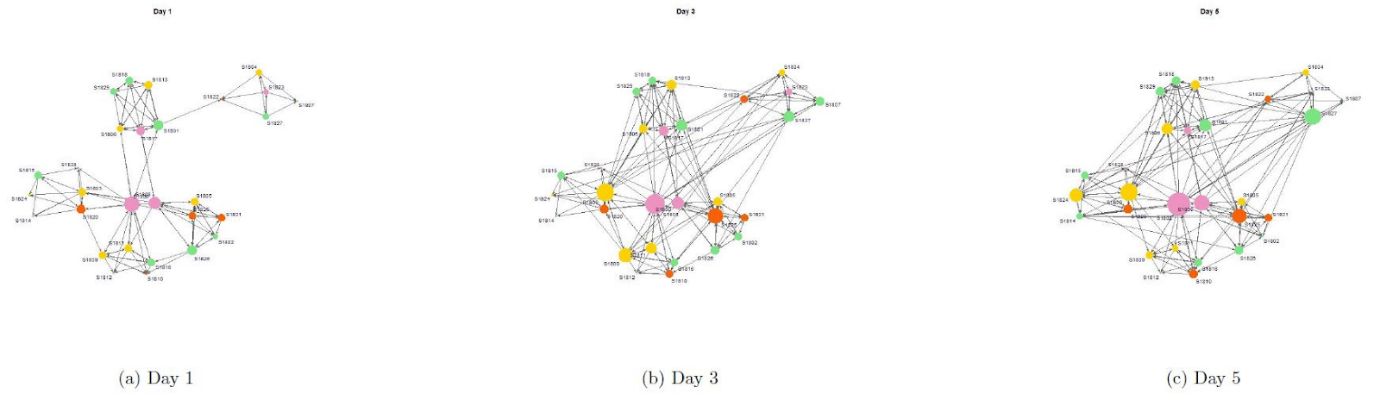  (c)Disciplines: Computer Science / Engineering / Data Science (pink), Medicine / Nursing (green), Psychology (yellow), and Public Health / Others (red) |

*S1-2 Fig. 2018 project based conversations by team(a), gender(b), and discipline(c)*

| 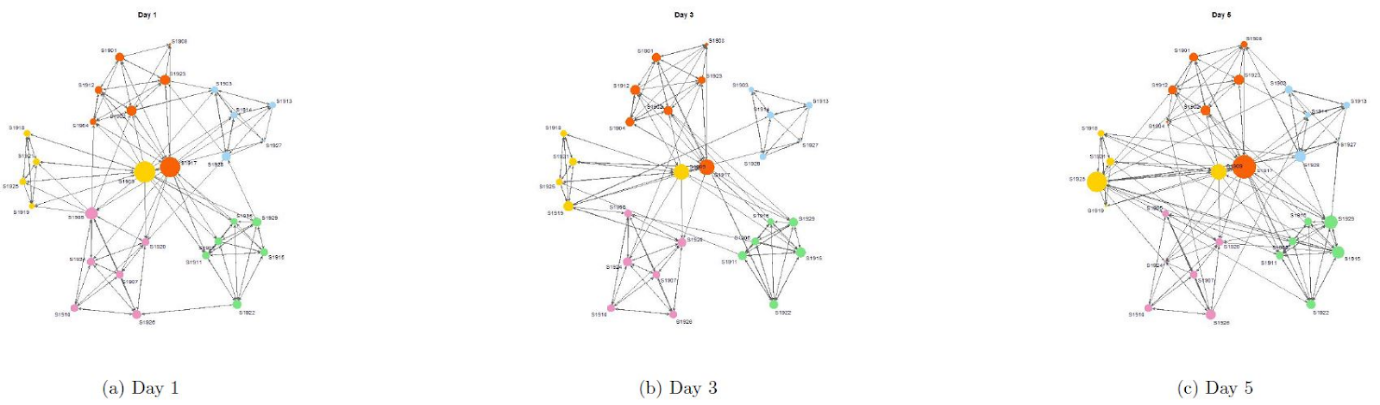  (a)Team: team 1 (pink), team 2 (green), team 3 (yellow), team 4 (red), and team 5 (sky blue) |
| --- |
| 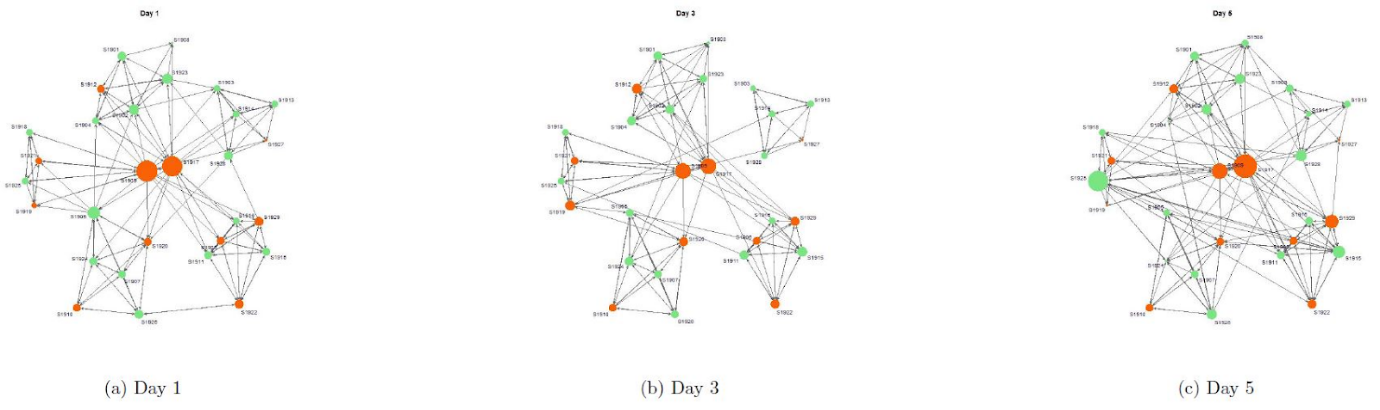  (b)Gender:  Female (green), Male (red), Decline to state (yellow) |
| 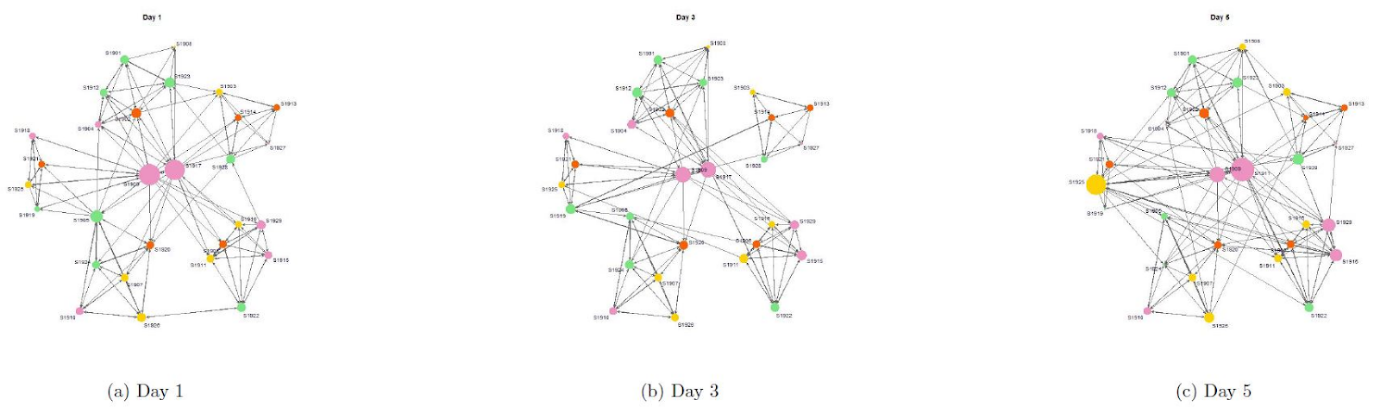  (c)Disciplines: Computer Science / Engineering / Data Science (pink), Medicine / Nursing (green), Psychology (yellow), and Public Health / Others (red) |

*S1-3 Fig. 2019 project based conversations by team(a), gender(b), and discipline(c)*
