# Supplementary material for: Relationships between changing communication networks and changing perceptions of psychological safety in a team science setting: Analysis with actor-oriented social network models
Source: PLoS One. 2022 Aug 31;17(8):e0273899. doi: 10.1371/journal.pone.0273899 (PMC9432705; doi:10.1371/journal.pone.0273899)
Supplement: S2 Appendix — (DOCX) [file pone.0273899.s003.docx]

**S2 Appendix. The goodness of fit plots**

Each Fig in this appendix includes goodness of fit plots for models 3 and 4 in each year. The sub-figures show how well the estimated models simulate network and behavior distributions, compared to the observed data in terms of 1) outdegree and indegree distributions and triad census for network structures and 2) behavior distribution. ‘p=0.05’ can be used as the rule of thumb to decide whether a model retrieves observed network and behavior data well or not [25]. In all three years, we observed that more than three of the four statistics had p-values larger than 0.05; only triad census in 2017 model 4 has its p-value of 0.049. These results support us to conclude that the final models were successfully able to replicate the observed data.

| 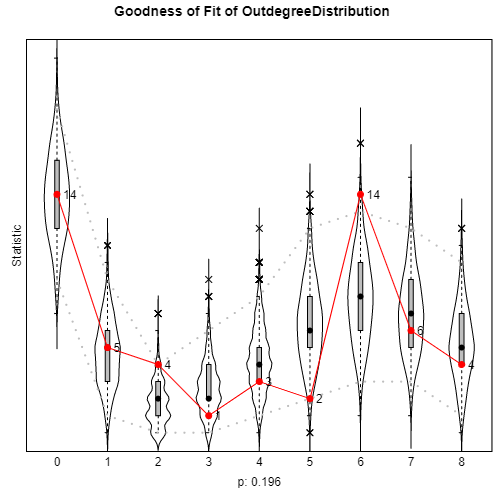  (a) Outdegree | 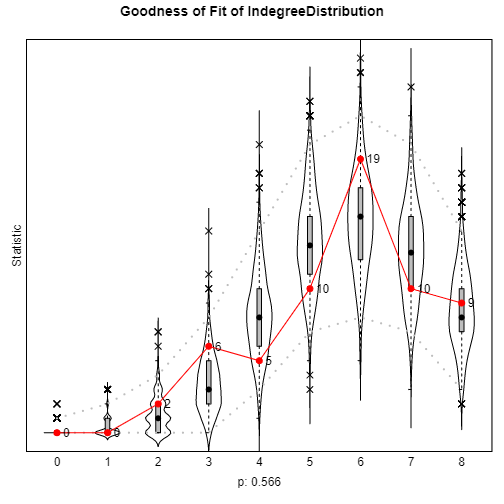  (b) Indegree |
| --- | --- |
| 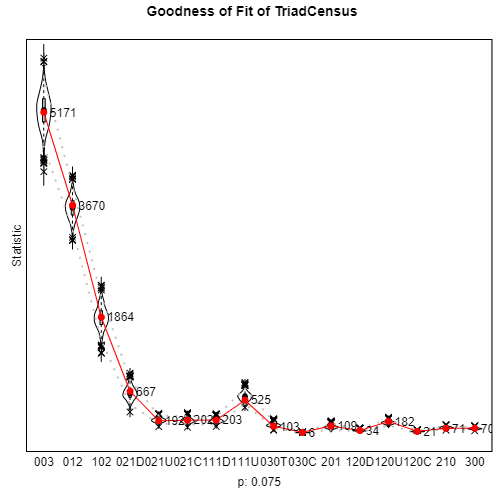  (c) Triad Census | 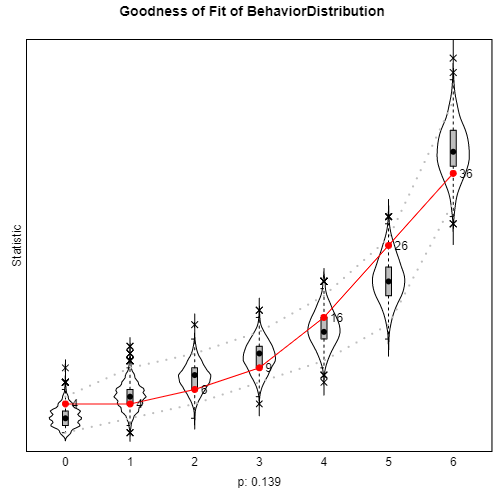  (d) Perception |

*S2-1 Fig. 2017 goodness of fit plot in model 3*

| 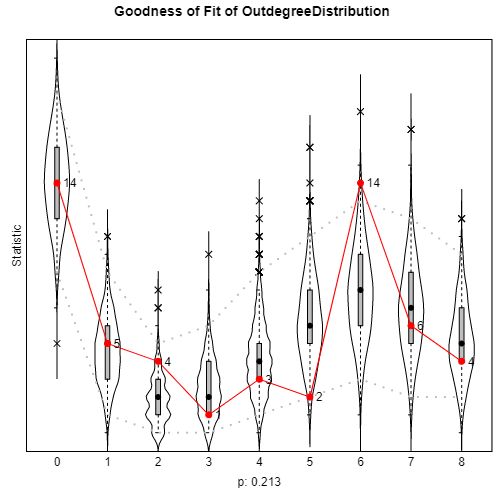  (a) Outdegree | 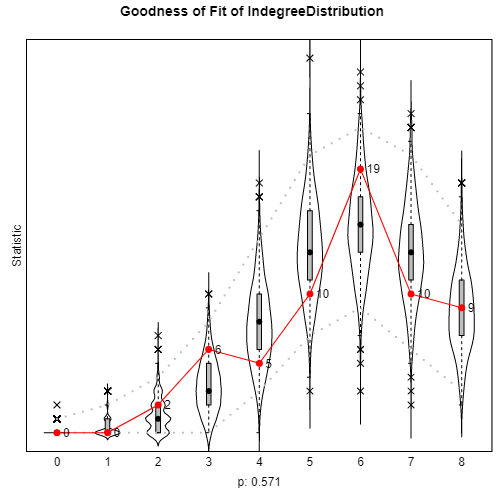  (b) Indegree |
| --- | --- |
| 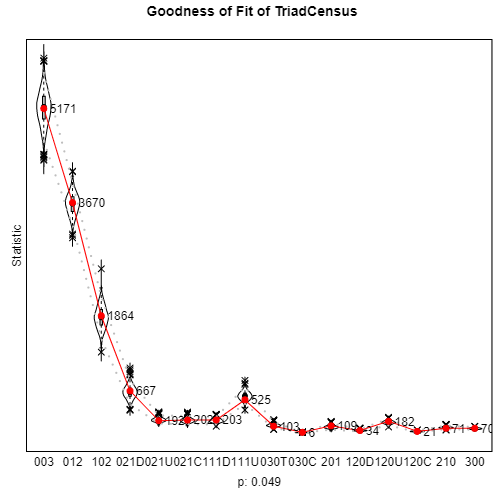  (c) Triad Census | 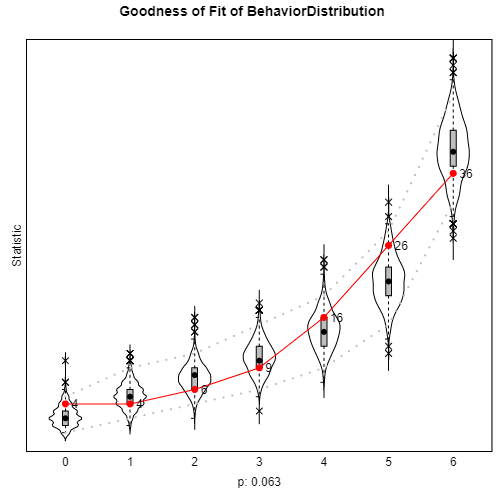  (d) Perception |

*S2-2 Fig. 2017 goodness of fit plot in model 4*

| 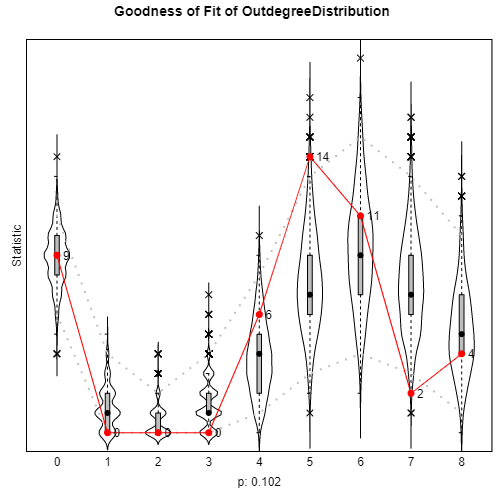  (a) Outdegree | 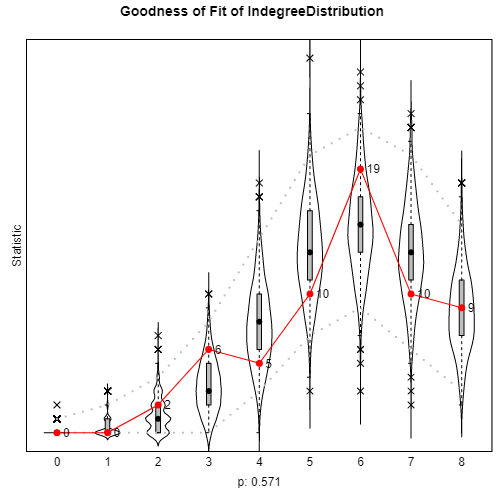  (b) Indegree |
| --- | --- |
| 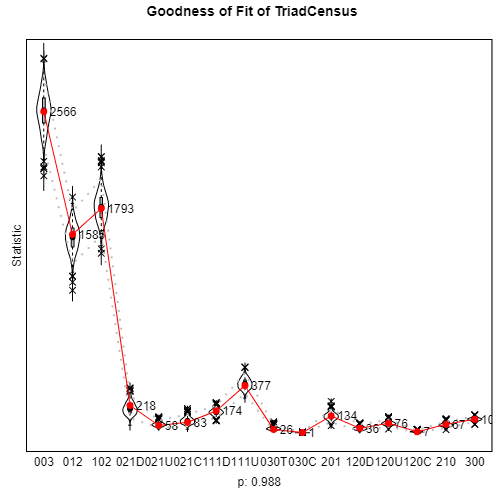  (c) Triad Census | 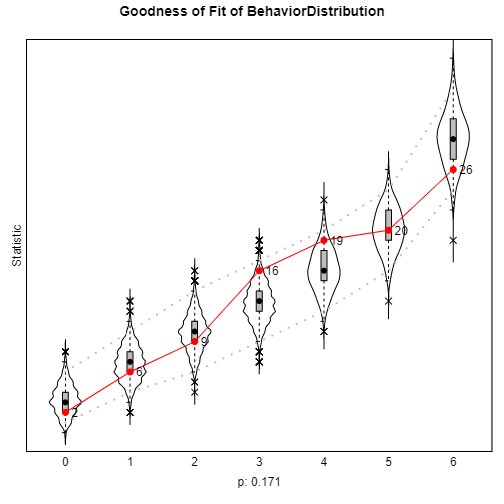  (d) Perception |

*S2-3 Fig. 2018 goodness of fit plot in model 3*

| 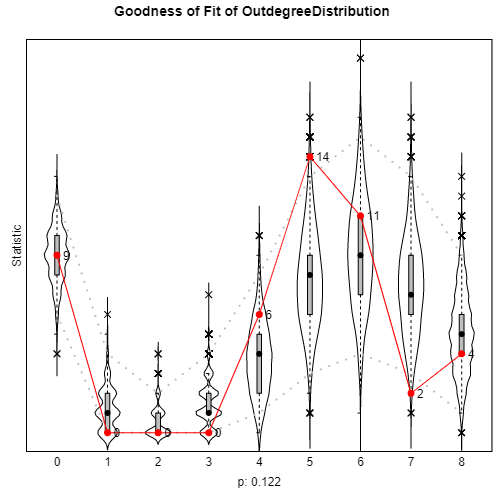  (a) Outdegree | 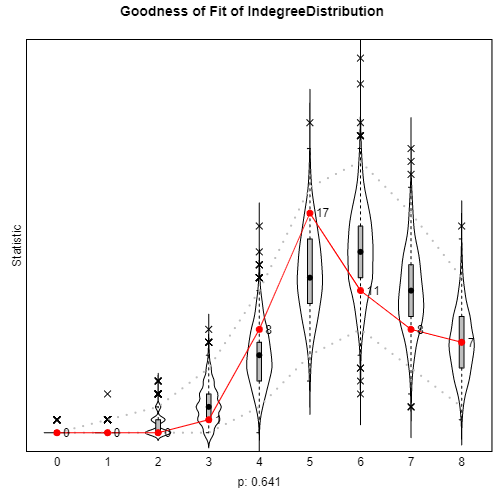  (b) Indegree |
| --- | --- |
| 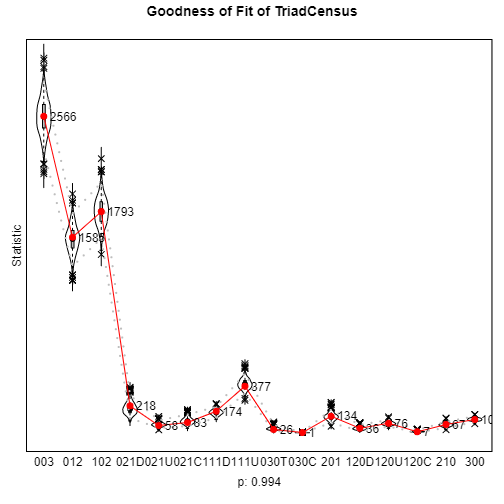  (c) Triad Census | 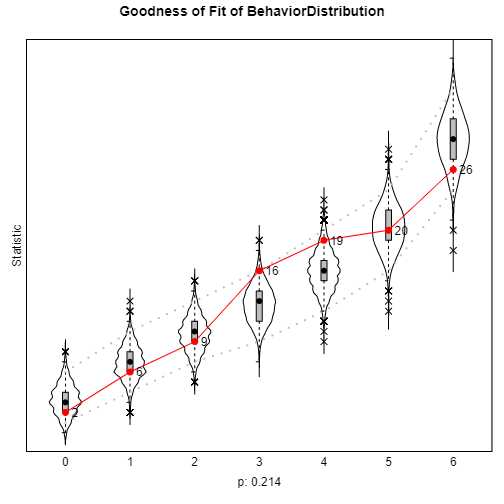  (d) Perception |

*S2-4 Fig. 2018 goodness of fit plot in model 4*

| 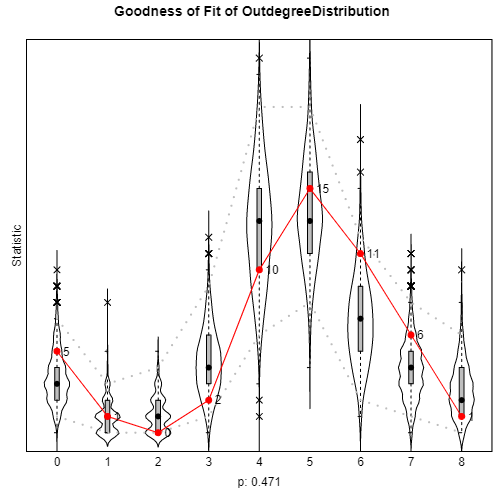  (a) Outdegree | 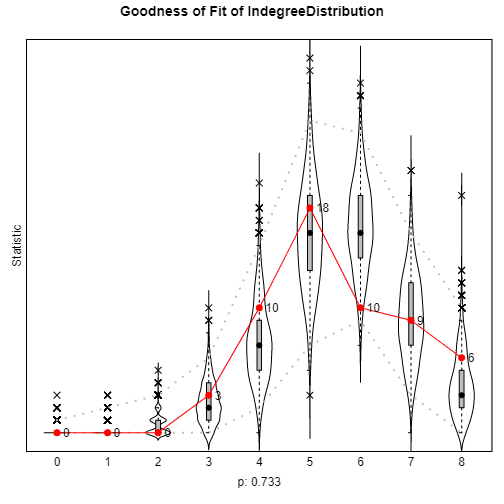  (b) Indegree |
| --- | --- |
| 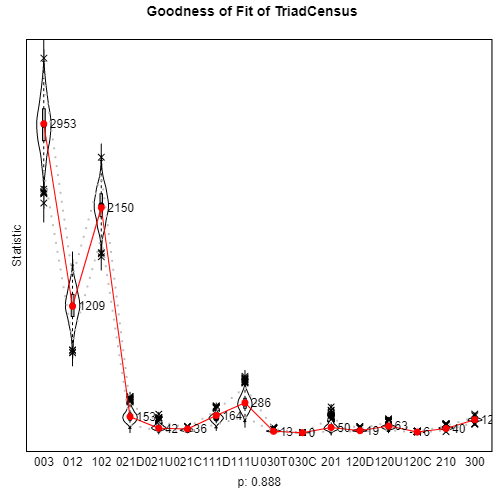  (c) Triad Census | 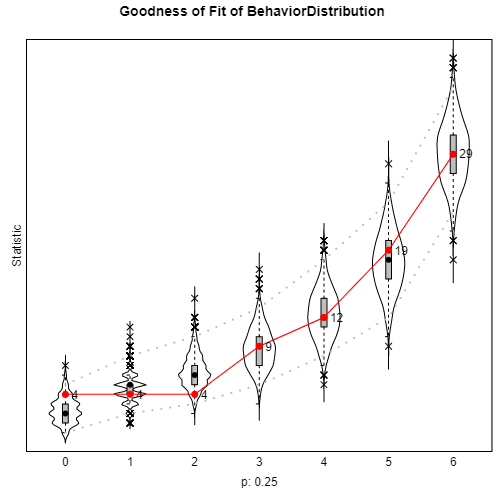  (d) Perception |

*S2-5 Fig. 2019 goodness of fit plot in model 3*

| 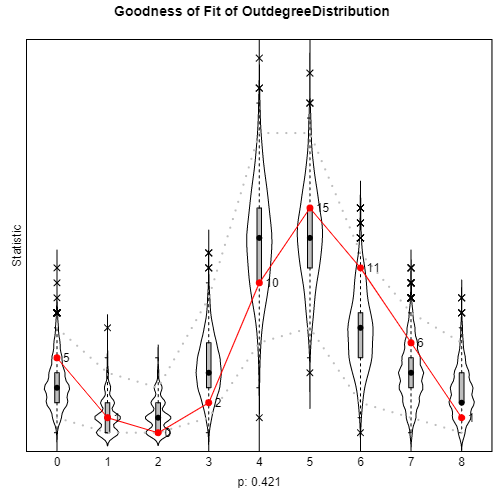  (a) Outdegree | 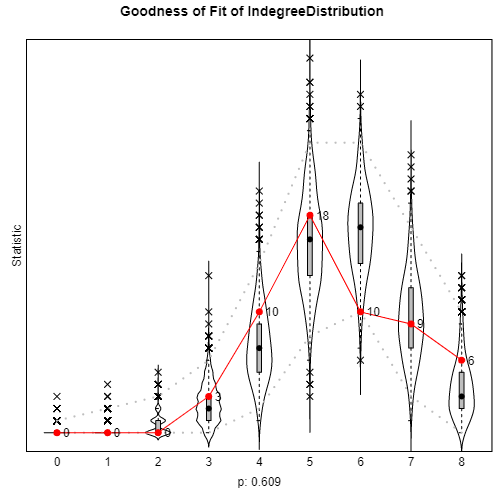  (b) Indegree |
| --- | --- |
| 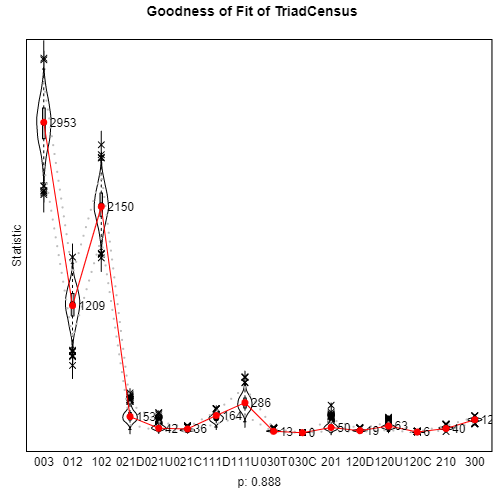  (c) Triad Census | 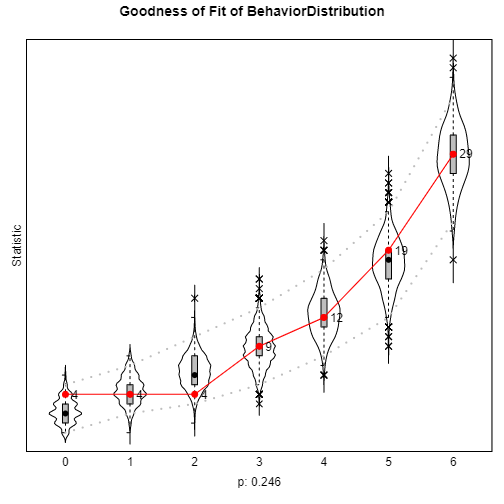  (d) Perception |

*S2-6 Fig. 2019 goodness of fit plot in model 4*
